# P2X1 enhances leukemogenesis through PBX3-BCAT1 pathways

**DOI:** 10.1038/s41375-022-01759-y

**Published:** 2022-11-23

**Authors:** Xiaoxiao He, Yilu Xu, Dan Huang, Zhuo Yu, Jing Yu, Li Xie, Ligen Liu, Ye Yu, Chiqi Chen, Jiangbo Wan, Yaping Zhang, Junke Zheng

**Affiliations:** 1 Hongqiao International Institute of Medicine, Shanghai Tongren Hospital, Key Laboratory of Cell Differentiation and Apoptosis of Chinese Ministry of Education, Faculty of Basic Medicine, Shanghai Jiao Tong University School of Medicine, Shanghai, 200025 China; 2 School of Basic Medicine and Clinical Pharmacy, China Pharmaceutical University, Nanjing, 211198 China; 3 Department of Hematology, Xinhua Hospital, Affiliated to Shanghai Jiao Tong University School of Medicine, Shanghai, 200092 China; 4 Research Unit of Stress and Cancer, Chinese Academy of Medical Sciences, Shanghai Cancer Institute, Renji hospital, Shanghai Jiao Tong University School of Medicine (SJTU-SM), Shanghai, 200127 China

**Keywords:** Acute myeloid leukaemia, Cancer stem cells

## Abstract

How bone marrow niches regulate leukemogenic activities of leukemia-initiating cells (LICs) is unclear. The present study revealed that the metabolic niche component, ATP, efficiently induced ion influx in LICs through its ligand-gated ion channel, P2X1. P2X1 deletion impaired LIC self-renewal capacities and resulted in an approximately 8-fold decrease in functional LIC numbers in a murine acute myeloid leukemia (AML) model without affecting normal hematopoiesis. P2X1 phosphorylation at specific sites of S387 and T389 was essential for sustaining its promoting effects on leukemia development. ATP-P2X1-mediated signaling upregulated the PBX3 level to transactivate BCAT1 to maintain LIC fates. P2X1 knockdown inhibited the proliferation of both human AML cell lines and primary cells. The P2X1 antagonist sufficiently suppressed AML cell proliferation. These results provided a unique perspective on how metabolic niche factor ATP fine-tunes LIC activities, which may benefit the development of strategies for targeting LICs or other cancer stem cells.

## Introduction

Acute myeloid leukemia (AML) is the most severe hematological malignancy and most commonly found in adults with high recurrence rates and mortality [1]. Leukemia-initiating cells (LICs) are a small cell population that is responsible for the initiation, progression, relapse and drug resistance of AML [2]. Although many intrinsic factors (i.e., transcription factors and epigenetic regulators) [3, 4] and extrinsic factors (bone marrow niche components) [5, 6] are required for LIC activities, there is still a lack of ideal targets for the eradication of LICs.

Increasing evidence shows that bone marrow niches play important roles in AML development [7, 8]. Many types of bone marrow niche cells and their secretory components, including growth factors, cytokines and metabolic products, are involved in LIC fate determinations [9, 10]. We recently also demonstrated that certain lipid metabolism regulators, such as ANGPTL2 and APOE, bind immune inhibitory receptors of LILRB2 and LILRB4, respectively, to significantly enhance AML-LIC self-renewal, migration or T cell suppression [11, 12]. These findings indicate that bone marrow metabolic niches may be tightly connected with LIC activities.

Adenosine triphosphate (ATP) mainly exists in cells and serves as a main energy carrier to support their biological behaviors. Various types of cells (e.g., neurons, platelets, lymphocytes and endothelial cells) also release ATP into the extracellular environment and contribute to many physiological and pathological activities [13, 14]. The extracellular ATP amounts in some solid tumors are increased to micromolar levels, and they promote tumorigenesis and chemoresistance by binding to purinergic receptors (P2Rs), including ligand-gated ion channels (P2Xs) and G protein-coupled membrane receptors (P2Ys) [15, 16]. P2X receptors (P2Xs) are cation-selective channels permeable to Na^+^, K^+^ or Ca^2+^, and ATP is the only known ligand for P2Xs, which are widely expressed in solid and hematological cancers [17, 18]. P2Xs contain seven family members (P2X1-7) with distinct ion channel properties and functions in response to ATP stimulation. For example, P2X1-4 can sensitively respond to extracellular ATP at nanomolar or micromolar levels, while P2X7 only responds to ATP at millimolar levels [19].

P2Xs have multifaceted roles in normal hematopoiesis or hematopoietic disorders. For example, P2X1 and P2X4 may be involved in the regulation of several fates of hematopoietic stem cells (HSCs) [20, 21]. P2X5 may be expressed in lymphoid malignancies, which can lead to LRH-1-specific cytotoxic T cell-mediated lysis [22]. P2X1- and P2X7-mediated signaling indirectly enhances the release of inflammatory factors from mesenchymal stem cells to regulate immune responses [23, 24]. P2X7 promotes AML development and impairs normal hematopoiesis via PBX3 signaling [25].

P2X1 is a nonselective ATP-gated and rapidly desensitizing ion channel expressed on smooth muscle cells [26], platelets [27], thymocytes [28] and neutrophils [29]. P2X1-mediated ion flux can be elicited at relatively low ATP levels (<10 nM), reach a peak within several seconds and last for more than 30 s. In contrast, high ATP levels trigger much faster ion flux than low ATP levels, reaching a peak within tens of milliseconds, and they are completely desensitized within seconds and quickly recover after desensitization [30, 31]. P2X1 plays important roles in male fertility, bladder contraction and platelet aggregation [32].

We recently demonstrated that ATP-P2X7-mediated signaling enhances AML development via the CREB-PHGDH pathway. Moreover, we noticed that several P2X members, except P2X7, such as P2X1, 4 and 5, were highly expressed in human AML samples, and P2X1 ranked top among them [33]. However, the detailed functions of the remaining P2X members, including P2X1, P2X4 and P2X5, in leukemogenesis remain largely unknown. Because P2X1 had the highest expression among all P2X members and was activated by extracellular ATP at the lowest level (nanomolar level), we evaluated its potential roles in sustaining LIC activities in the present study.

## Results

### P2X1 is highly expressed in LICs and required for their leukemogenic activities

**Fig. 1:**
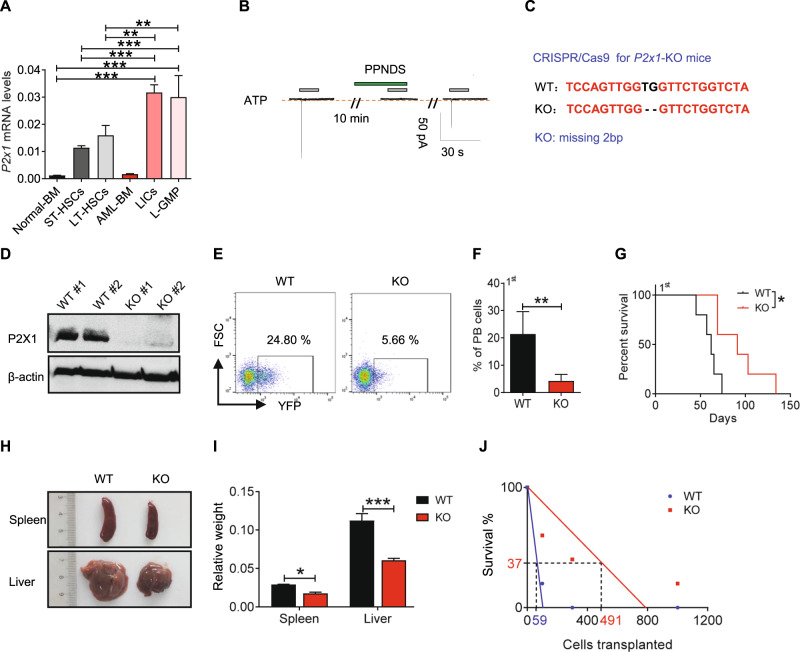
P2X1 is highly enriched in LICs and required for leukemogenesis. **A** The mRNA levels of *P2x1* in normal mouse bone marrow cells (Normal-BM), Lin^-^Sca-1^+^c-Kit^+^CD34^+^Flk2^+^ ST-HSCs, Lin^-^Sca-1^+^c-Kit^+^CD34^-^Flk2^-^ LT-HSCs, YFP^+^ leukemia cells (AML-BM), Mac-1^+^c-Kit^+^ LICs and Lin^-^Sca-1^-^c-Kit^+^CD34^+^CD16/32^-^ L-GMP cells were measured by qRT–PCR (*n* = 3). **B** P2X1-mediated ion influx in murine Mac-1^+^c-Kit^+^ LICs was measured after sequential treatments with extracellular ATP and the PPNDS P2X1 antagonist by whole-cell patch-clamp recording. Data from three independent experiments. **C** Details of the nucleotide base sequences deleted in *P2x1* knockout (*P2x1*-KO or *P2x1*-null) mice using the CRISPR-Cas9 strategy. **D** P2X1 protein levels in WT (WT #1-#2) and *P2x1*-KO (*P2x1*-KO #1-#2) BM cells were measured by western blot analysis. **E** Representative flow cytometric analysis of leukemia cells (YFP^+^) in the peripheral blood of recipient mice upon primary transplantation. **F** Quantification of the data shown in **E** (*n* = 5). **G** The overall survival of recipient mice transplanted with WT or *P2x1*-KO MLL-AF9^+^ BM cells upon primary transplantation (*n* = 5). **H** Representative images of the size of the spleens and livers of recipients upon primary transplantation. **I** Quantification of the data shown in **H** (*n* = 3). **J** Limiting dilution assays were performed with WT and KO BM YFP^+^ AML cells from the primary transplantation at the indicated cell doses, and functional LIC frequencies were calculated. Data are presented as the mean ± SD. One-way ANOVA with Tukey’s multiple comparison test (**A**), student’s two-tailed unpaired *t* test (**F**), log-rank test (**G**) and two-way ANOVA with Sidak’s multiple comparison test (**I**) were used for the comparison of statistical significance (*P < 0.05; **P < 0.01; and ***P < 0.001).

To evaluate the role of P2X1 in AML development, we first measured *P2x1* mRNA levels in immunophenotypic Lin^–^Sca-1^+^c-Kit^+^CD34^-^Flt3^-^ LT-HSCs and Mac-1^+^c-Kit^+^ LICs or Lin^–^Sca-1^+^c-Kit^+^CD34^+^CD16/CD32^+^ L-GMP cells (a population more highly enriched in LICs, Fig. 1A). We further demonstrated that immunophenotypic LICs or L-GMPs had approximately 20-fold higher expression levels of *P2x1* than total AML cells, normal BM cells or HSCs (Fig. 1A), which was indicating that P2X1 may play key roles in leukemogenesis. Consistently, in vitro ATP simulation efficiently elicited ion flux in murine LICs, which was completely blocked by PPNDS, a selective P2X1 antagonist (Fig. 1B). To evaluate the potential roles of P2X1 in AML development, we knocked down *P2x1* in murine AML cells using shRNAs (Fig. S1A), and demonstrated that leukemia development was significantly delayed in the recipient mice receiving *P2x1*-knockdown AML cells than WT counterparts as revealed by reduced leukemia cell frequencies in the peripheral blood (Figs. S1B, C) and prolonged overall survival of leukemic mice (47 or 56 vs. 36 days, Fig. S1D).

We then generated *P2x1* knockout mice by the CRISPR-Cas9 technique with the deletion of 2 bp in exon 1 (Fig. 1C). P2X1 protein levels were undetected in total BM cells (Fig. 1D). We next established an MLL-AF9-induced murine AML model as previously described [34], and found that P2X1 deletion led to a notable delay in leukemia development in recipient mice receiving MLL-AF9-overexpressing *P2x1*-null Lin^-^ BM cells as revealed by decreased YFP^+^ leukemic cell frequencies in the peripheral blood (4.3% vs. 21.4%, Fig. 1E, F and S1E, F), extended overall survival (91 vs. 62 days, Fig. 1G) and reduced leukemic cell infiltration in the spleens and livers of transplant mice (Fig. 1H, I and S1G). Limiting dilution assays with YFP^+^ AML cells from primary transplant recipients revealed that the frequency of functional WT LICs was 1 in 59, which was approximately 8.3-fold higher than that of *P2x1*-null control cells (1 in 491; Fig. 1J and Table S1).

In addition, we noticed that several P2X members, including P2X1, 4, 5 and 7, were highly expressed in AML cells. We also evaluated the roles of the remaining two P2X members in leukemogenesis, and demonstrated that P2X4 and P2X5 were not required for the maintenance of LIC leukemogenic activities (Fig. S1H, I), but P2X1 and P2X7 could enhance leukemogenic activities [33]. Interestingly, no significant difference in the percentages of LT-HSCs was observed between WT and *P2x1*-null mice (Fig. S1J, K). *P2x1*-null LT-HSCs also had repopulation abilities comparable to their WT counterparts (Fig. S1L–N). These data indicated that P2X1 supports leukemia development while sustaining normal hematopoiesis, which may be an ideal target for leukemia treatments.

### P2X1 is important for the self-renewal of LICs

**Fig. 2:**
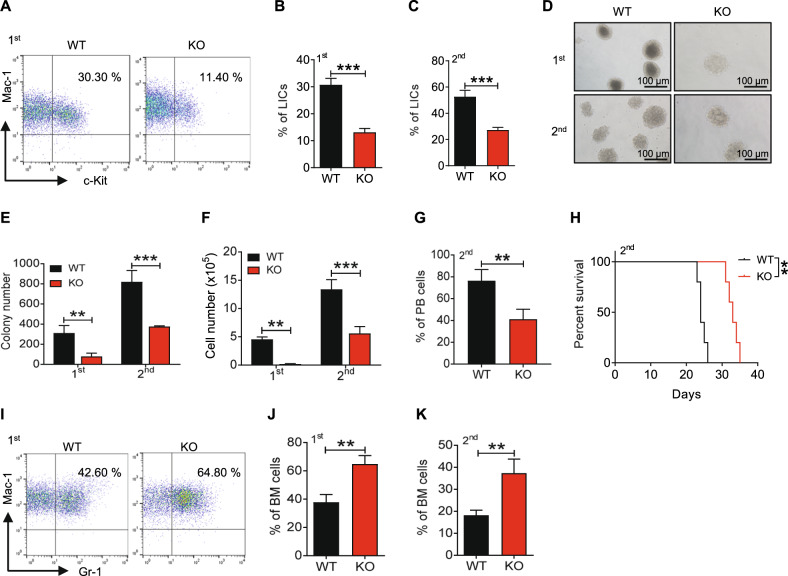
P2X1 promotes self-renewal and inhibits the differentiation of LICs. **A** Representative flow cytometric analysis of Mac-1^+^c-Kit^+^ LICs in recipient mice upon primary transplantation. **B**, **C** The frequencies of WT and *P2x1*-KO Mac-1^+^c-Kit^+^ LICs from recipients upon primary (**B**) and secondary (**C**) transplantation (*n* = 3). **D** Representative images of colonies derived from WT and *P2x1*-KO YFP^+^ AML cells during the 1st and 2nd plating. **E**, **F** Colony numbers (**E**) and derived total cell counts (**F**) from WT and *P2x1*-KO YFP^+^ AML cells during the 1st and 2nd plating (*n* = 3). **G** Quantification of the frequencies of leukemia cells (YFP^+^) in the peripheral blood of recipient mice 3 weeks after transplantation (*n* = 5). **H** Overall survival was determined for the leukemic mice shown in **G** (*n* = 5). **I** Representative flow cytometric analysis of Mac-1^+^Gr1^+^ cells in recipient mice upon primary transplantation. **J**, **K** The frequencies of WT and *P2x1*-KO Mac-1^+^Gr1^+^ cells from the recipients upon primary (**J**) and secondary (**K**) transplantation (*n* = 3). Data are presented as the mean ± SD. Student’s two-tailed unpaired *t* test (**B**, **C**, **G**, **J** and **K**), log-rank test (**H**) and two-way ANOVA with Sidak’s multiple comparison test (**E** and **F**) were used for the comparison of statistical significance (**P < 0.01; and ***P < 0.001).

To further evaluate the role of P2X1 in sustaining LIC activities, we performed a serial transplantation with *P2x1*-null AML cells and revealed that the immunophenotypic Mac-1^+^c-Kit^+^ *P2x1*-null LIC frequency was notably decreased to 30.7% or 13.1% of WT LICs upon primary (Fig. 2A, B) or secondary transplantation (Fig. 2C), respectively. *P2x1*-null leukemic cells gave rise to significantly fewer colony numbers (Fig. 2D, E) and derived total cell counts (Fig. 2F) during the 1st and 2nd plating. Moreover, *P2x1*-null AML cells had impaired self-renewal abilities compared to WT controls as evidenced by decreased leukemic cell frequencies in the peripheral blood and significantly extended overall survival in the secondary transplantation assay (Fig. 2G, H). Consistently, the leukemic mice that received *P2x1*-knockdown AML cells had significantly longer overall survival than those that received scrambled cells (Fig. S2A, B). In addition, a significant difference in differentiation was observed after P2X1 deletion during the 1st and 2nd transplantation as measured by Mac-1/Gr-1 staining (Gr-1 level indicates the extent of differentiation, Fig. 2I–K). Meanwhile, we demonstrated that there was no difference in cell cycle between WT and *P2x1*-null LICs (Fig. S2C, D). However, there were much higher frequencies of early/late apoptotic cells in *P2x1*-null LICs compared to WT control cells (Fig. S2E, F). These results indicate that P2X1 may also promote AML development by suppressing LIC apoptosis.

### P2X1 regulates LIC activities via BCAT1-mediated branched chain amino acid metabolism

**Fig. 3:**
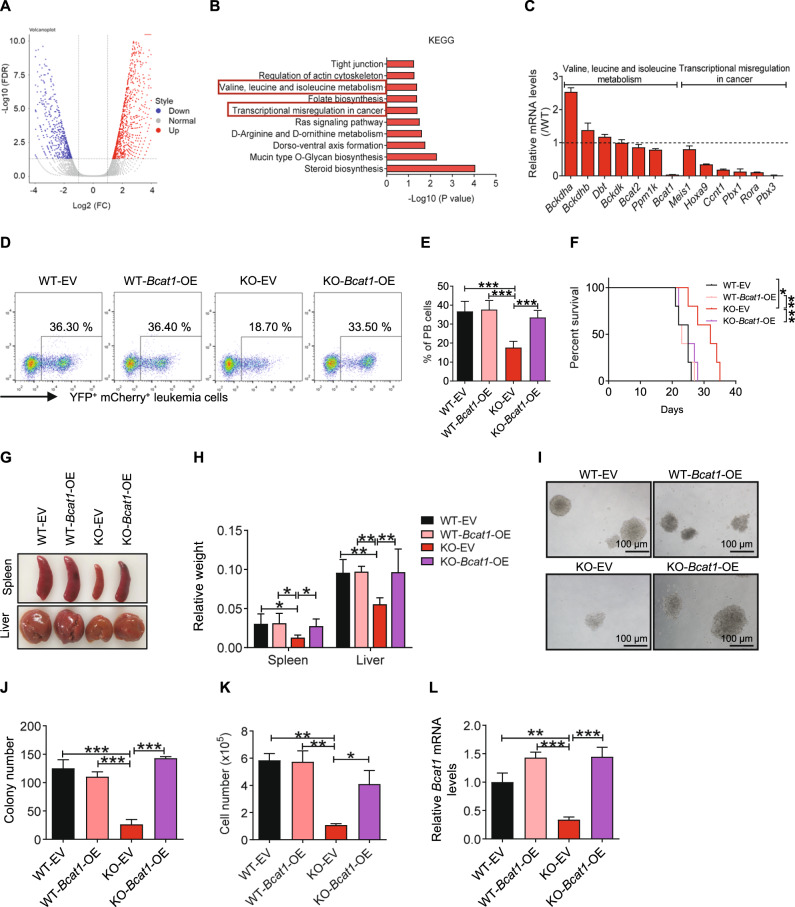
P2X1 regulates LIC activities via BCAT1-mediated branched chain amino acid metabolism. **A** Volcano plots showing differentially expressed genes in WT and *P2x1*-KO Mac-1^+^c-Kit^+^ LICs. **B** KEGG pathway analysis was performed using mRNA-sequencing data from WT or *P2x1*-KO Mac-1^+^c-Kit^+^ LICs. **C** Potential candidates related to the “valine, leucine and isoleucine (branched chain amino acid, BCAA) metabolism” and “transcriptional misregulation in cancer” pathways were examined in WT and *P2x1*-KO Mac-1^+^c-Kit^+^ LICs by qRT–PCR (*n* = 3). **D** Representative flow cytometric analysis of YFP^+^mCherry^+^ leukemia cells (The markers for the indication of leukemia cells were YFP^+^ and mCherry^+^, which were the tags for the MSCV-MLL-AF9-YFP plasmid and MSCV-mCherry overexpression plasmid) in the peripheral blood of recipient mice transplanted with WT, *P2x1*-KO, *Bcat1*-overexpressing WT or *P2x1*-KO leukemia cells. **E** Quantification of the data shown in D (*n* = 5). **F** The overall survival was compared among the mice transplanted with WT, *P2x1*-KO, *Bcat1*-overexpressing WT or *P2x1*-KO leukemia cells (*n* = 5). **G** Representative images of the spleen and liver sizes of recipient mice transplanted with WT, *P2x1*-KO, *Bcat1*-overexpressing WT or *P2x1*-KO leukemia cells. **H** Quantification of the data shown in **G** (*n* = 3). **I** Representative images of colonies derived from WT, *P2x1*-KO, *Bcat1*-overexpressing WT or *P2x1*-KO YFP^+^mCherry^+^ AML cells. **J**, **K** Quantification of the colony numbers (**J**) and derived total cell counts (**K**) in **I** (*n* = 3). **L** The *Bcat1* mRNA levels in BM cells from recipient mice transplanted with WT, *P2x1*-KO, *Bcat1*-overexpressing WT or *P2x1*-KO AML cells were measured by qRT–PCR (*n* = 3). Data are presented as the mean ± SD. One-way ANOVA with Tukey’s multiple comparison test (**E**, **J**, **K** and **L**), log-rank test (**F**) and two-way ANOVA with Sidak’s multiple comparison test (**H**) were used for the comparison of statistical significance (*P < 0.05; **P < 0.01; and ***P < 0.001).

To understand the potential downstream targets of P2X1 in the regulation of LIC self-renewal capacities, we performed RNA-sequencing analysis of WT and *P2x1*-null immunophenotypic Mac-1^+^c-Kit^+^ LICs. Approximately 1383 upregulated and 864 downregulated genes were observed after P2X1 deletion (Fig. 3A). Among the top 10 significantly downregulated pathways according to the Kyoto Encyclopedia of Genes and Genome (KEGG) analyses, P2X1 was associated with the following pathways: valine, leucine and isoleucine metabolism (or branched chain amino acid (BCAA) metabolism); folate biosynthesis; and transcriptional misregulation in cancer (Fig. 3B). We then examined the expression levels of several key enzymes involved in BCAA catabolism (such as *Bcat1*, *Bcat2*, *Bckdha*, *Bckdhb*, *Bckdk*, *Ppm1k* and *Dbt*) and transcription factors (such as *Meis1*, *Hoxa9*, *Ccnt1*, *Pbx1*, *Rora* and *Pbx3*) that have been reported to be involved in the regulation of LIC activities from RNA-sequencing data. Interestingly, several genes, including *Bcat2*, *Bckdhb*, *Bcat1*, *Hoxa9*, *Meis1*, *Ccnt1*, *Pbx3*, *Rora* and *Pbx1*, were significantly decreased in *P2x1*-null LICs (Fig. S3A). Quantitative RT–PCR (qRT-PCR) further showed that *Bcat1* (related to BCAA metabolism) or *Hoxa9*, *Ccnt1*, *Pbx1*, *Rora* and *Pbx3* (related to transcriptional misregulation in cancer) were significantly reduced in *P2x1*-null LICs (Fig. 3C and Table S2), suggesting that these genes may act as downstream targets of P2X1.

BCAT1-mediated BCAA metabolism has been reported to be essential in sustaining LIC activities via enhanced mTOR signaling pathways [35]. PBX1 and PBX3 are two key transcription factors involved in the maintenance of stem cell activities and tumorigenesis [36, 37]. Therefore, we first overexpressed Bcat1 in *P2x1*-null AML cells and showed that ectopic overexpression of *Bcat1* almost completely rescued the P2X1 loss of function in recipient mice as evidenced by increased leukemic cell frequencies in the peripheral blood (Fig. 3D, E), reduced overall survival (Fig. 3F) and enhanced infiltration in spleens and livers (Fig. 3G, H). *Bcat1* overexpression had no effect on WT AML cell growth in vivo (Fig. 3D–H). An in vitro colony formation assay also revealed that overexpression of Bcat1 in *P2x1*-null AML cells resulted in increased colony numbers and total derived cell numbers (Fig. 3I–K). The mRNA expression levels of *Bcat1* in WT and *P2x1*-null AML cells were validated by qRT–PCR (Fig. 3L). Consistently, ectopic overexpression of *Bcat1* in *P2x1* knockdown AML cells decreased the leukemia cell frequencies in the peripheral blood (Fig. S3B, C) and overall survival of recipient mice (Fig. S3D). We also measured the expression levels of LIC associated genes *Meis1, Hoxa9, Pbx1 and Pbx3* in Mac-1^+^c-Kit^+^ LICs from recipient mice transplanted with WT, *P2x1*-null, *Bcat1*-overexpressing WT or *P2x1*-null AML cells by qRT-PCR. Interestingly, *Bcat1* overexpression can restore the expression of *Meis1 and Hoxa9*, but not *Pbx1* and *Pbx3* (Fig. S3E).

To understand the potential roles of P2X1 in LIC activities, we further examined the potential candidates involved in LIC self-renewal and differentiation. Several genes that have been reported to be related to the self-renewal (*Creb, Hoxb4, Hoxa9, Myb, Cbx5, Meis1, Bmi1, Hmgb3 and Pbx1*) of AML cells were indeed decreased in the *P2x1*-null LICs, whereas the differentiation gene expression signatures (*Gfi1b, Gata2, Cebpe and Cebpg)* were increased (Fig. S3F) [4, 38–41]. Consistently, qRT-PCR further demonstrated that most of the self-renewal genes, including S*tat5b, Creb, Hoxa9, Myb, Cbx5, Meis1, Bmi1, Humg3 and Pbx1*, were significantly downregulated in the *P2x1*-null LICs. In contrast, the differentiation related genes, including *Gfi1b, Gata2, Cebpg, Klf4 and Pu.1*, were significantly upregulated in the *P2x1*-null LICs (Fig. S3G).

### PBX3 transactivates BCAT1 expression to sustain LIC activities

**Fig. 4:**
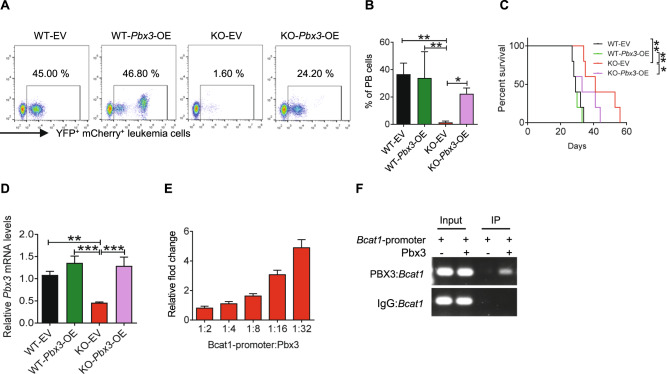
PBX3 transactivates *BCAT1* expression to sustain LIC activities. **A** Representative flow cytometric analysis of YFP^+^mCherry^+^ leukemia cells (The markers for the indication of leukemia cells were YFP^+^ and mCherry^+^, which were the tags for the MSCV-MLL-AF9-YFP plasmid and MSCV-mCherry overexpression plasmid) in the peripheral blood of recipient mice transplanted with WT, *P2x1*-KO, *Pbx3*-overexpressing WT or *P2x1*-KO leukemia cells. **B** Quantification data in **A** (*n* = 5). **C** The overall survival was compared among the mice transplanted with WT, *P2x1*-KO, *Pbx3*-overexpressing WT or *P2x1*-KO leukemia cells (*n* = 5). **D** The mRNA levels of *Pbx3* in BM cells of recipient mice transplanted with WT, *P2x1*-KO, *Pbx3*-overexpressing WT or *P2x1*-KO AML cells were measured by qRT–PCR (*n* = 3). **E** A luciferase reporter assay was used to evaluate PBX3 transcriptional activities on *Bcat1* (*n* = 3). Data from three independent experiments. **F** The binding of PBX3 to the *Bcat1* promoter region was determined by a ChIP assay. Data are presented as the mean ± SD. One-way ANOVA with Tukey’s multiple comparison test (**B** and **D**) and log-rank test (**C**) were used for the comparison of statistical significance (*P < 0.05; **P < 0.01; and ***P < 0.001).

To further delineate the upstream targets of BCAT1, we evaluated several potential signaling pathways downregulated in *P2x1*-null LICs, such as transcriptional misregulation in cancer, and we found that the *Pbx3* mRNA level in *P2x1*-null LICs was significantly decreased to 10% of that in WT LICs (Fig. 3C). We then overexpressed *Pbx3* in *P2x1*-null AML cells and demonstrated that its ectopic expression enhanced AML progression in recipient mice as indicated by increased leukemic cell frequencies in the peripheral blood (Fig. 4A, B), reduced overall survival (Fig. 4C, D) and enhanced infiltration in spleens and livers (Fig. S4A). Consistently, *Pbx3*-overexpressing *P2x1*-null AML cells had enhanced clonogenic capacities as well as increased colony numbers and total derived leukemic cells (Fig. S4B–D). We also measured the *Bcat1* mRNA levels in WT, *P2x1*-null, *Pbx3*-overexpressing WT or *P2x1*-null leukemia cells and showed that the *Bcat1* mRNA level was significantly downregulated in *P2x1*-null AML cells, while increased in *Pbx3*-overexpressing leukemic cells (Fig. S4E).

To determine potential connections between PBX3 and BCAT1, we performed a dual-luciferase reporter assay and found that PBX3 directly bound the *Bcat1* promoter to transactivate its expression in a dose-dependent manner (Fig. 4E), which was further confirmed by a ChIP experiment (Fig. 4F). As mentioned above, BCAT1-mediated BCAA metabolism may be involved in the regulation of mTOR signaling and mitochondrial function, we then examined the mitochondrial metabolisms upon *P2x1* deletion. Interestingly, P2X1 loss could cause the decrease in the mitochondrial membrane potential and ROS levels (Fig. S4F, G). Consistently, *P2X1*-null AML cells had much lower levels of glycolysis and oxidative phosphorylation as evidenced by the notable reduction in ATP, ECARs, and OCRs than WT counterparts (Fig. S4H–J).

### P2X1 phosphorylation sites at S387 and T389 are critical for the leukemogenic activities of LICs

**Fig. 5:**
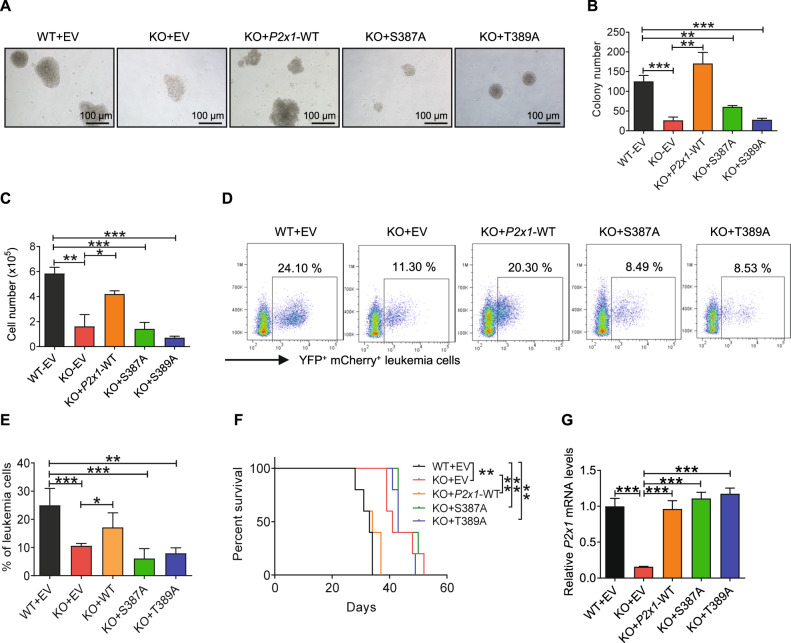
P2X1 phosphorylation sites at S387 and T389 are critical for leukemogenic activities. **A** Representative images of colonies derived from WT and *P2x1*-KO AML cells ectopically expressing empty vector (EV), WT or mutant *P2x1* (S387A or T389A). **B**, **C** Quantification of the colony numbers (**B**) and derived total cell counts (**C**) shown in **A** (*n* = 3). **D** Representative flow cytometric analysis of YFP^+^mCherry^+^ leukemia cells (The markers for the indication of leukemia cells were YFP^+^ and mCherry^+^, which were the tags for the MSCV-MLL-AF9-YFP plasmid and MSCV-mCherry overexpression plasmid) in the peripheral blood of recipient mice transplanted with WT, *P2x1*-KO AML cells ectopically expressing empty vector (EV), WT or mutant *P2x1* (S387A or T389A). **E** Quantification of data shown in **D**. One representative out of two independent experiments with *n* = 5 mice per group is shown. **F** The overall survival was compared among the mice transplanted with WT, *P2x1*-KO AML cells ectopically expressing empty vector (EV), WT or mutant *P2x1* (S387A or T389A) (*n* = 5). **G** The *P2x1* mRNA levels in BM cells of recipient mice transplanted with WT, *P2x1*-KO AML cells ectopically expressing empty vector (EV), WT or mutant *P2x1* (S387A or T389A) were measured by qRT–PCR (*n* = 3). Data are presented as the mean ± SD. One-way ANOVA with Tukey’s multiple comparison test (**B**, **C**, **E** and **G**) and log-rank test (**F**) were used for the comparison of statistical significance (*P < 0.05; **P < 0.01; and ***P < 0.001).

Because P2X1 can be phosphorylated at several phosphorylation sites [42], including S387, S388 and T389, we investigated whether these phosphorylation sites play a role in AML development by mutating the abovementioned P2X1 phosphorylation sites (Table S2) and evaluating their potential effects on leukemogenesis. Among the S387A, S388A and T389A mutants, neither S387A nor T389A rescued the impaired leukemogenic activities of the *P2x1-*knockdown murine AML cell line, C1498 (Fig. S5A, B). The colony formation assay showed that overexpression of both *P2x1*-S387A and *P2x1*-T389A in *P2x1*-null AML cells did not affect in vitro cell growth compared to *P2x1*-null control cells (Fig. 5A–C). In vivo transplantation experiments showed that the recipient mice receiving *P2x1*-null AML cells with ectopic expression of either *P2x1*-S387A or the *P2x1*-T389A mutant had similar overall survival to the mice receiving *P2x1*-null AML cells (Fig. 5D–F). The overexpression levels of *P2x1*-WT, *P2x1*-S387A and *P2x1*-T389A in *P2x1*-null AML were validated by qRT–PCR (Fig. 5G). In contrast, overexpression of the *P2x1*-S388A mutant and *P2x1*-WT control in *P2x1*-null AML cells had a similar effect on cell proliferation as indicated by the in vitro colony formation assay (Fig. S5C–E) and the in vivo transplantation assay (Fig. S5F–H).

### P2X1 is required for the proliferation of human AML cells

**Fig. 6:**
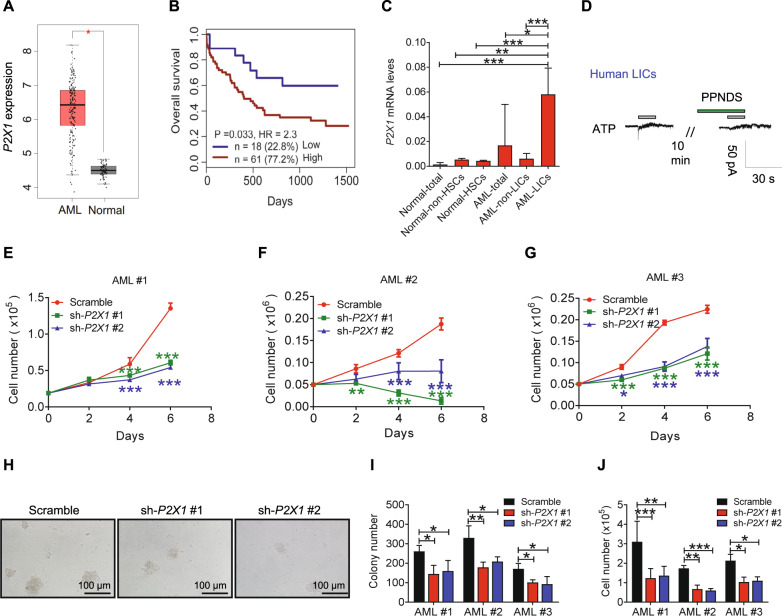
P2X1 is required for the proliferation of human AML cells. **A** The *P2X1* mRNA levels were analyzed in AML cells and normal BM cells from TCGA databases (AML, *n* = 173; normal, *n* = 70). **B** Relationship between *P2X1* mRNA levels and overall survival in AML patients from the Genomicscape dataset. **C** The mRNA levels of *P2X1* in human normal cord blood cells (Normal-total), normal CD34^-^ cells (Normal-non-HSCs), normal Lin^-^CD34^+^CD38^-^CD45RA^-^CD90^+^ HSCs (Normal-HSCs), human primary AML cells (AML-total), CD34^-^ cells (AML-non-LICs) and human primary CD34^+^ AML-LICs (AML-LICs) were measured by qRT–PCR (*n* = 3). **D** The P2X1-mediated ion influx in human CD34^+^ LICs was measured after sequential treatments with extracellular ATP and the PPNDS P2X1 antagonist by whole-cell patch-clamp recording (*n* = 3). **E**–**G** The numbers of human primary AML cells were counted at the indicated days upon *P2X1* knockdown by shRNAs (sh-*P2X1* #1 and #2) and a scrambled control. Three patient samples were used for the indicated experiments (AML #1-#3) (n = 3). (**H**) Representative images of colonies derived from human AML cells after *P2X1* knockdown by shRNAs (sh-*P2X1* #1 and #2) or a scrambled control. **I**, **J** Colony numbers (**I**) and derived total cell counts (**J**) were counted in human AML cells after *P2X1* knockdown by shRNAs (sh-*P2X1* #1 and #2) and scrambled controls (*n* = 3). Data are presented as the mean ± SD. Student’s two-tailed unpaired *t* test (**A**), one-way ANOVA with Tukey’s multiple comparison test (**C**) and two-way ANOVA with Sidak’s multiple comparison test (**E**–**G**, **I** and **J**) were used for the comparison of statistical significance (*P < 0.05; **P < 0.01; and ***P < 0.001).

We have previously reported that *P2X1* has the highest mRNA expression levels in AML cells among all P2X members according to TCGA databases [33]. We analyzed TCGA data and showed that *P2X1* mRNA levels in human AML cells were significantly higher than those in normal BM cells (Fig. 6A), which were adversely correlated with patient overall survival (TCGA and http://www.genomicscape.com) (Fig. S6A and Fig. 6B). *P2X1* was highly expressed in several human AML cell lines, especially in FAB-M5-type AML cells, including THP-1 (M5), U937 (M5) and MV4-11 (M5) cells (Fig. S6B). AML cell lines from other FAB AML types, such as HL60 (M3) and NB4 (M3) cells, had relatively low *P2X1* mRNA levels. We knocked down *P2X1* in these cell lines using two shRNAs (Fig. S6C) and demonstrated that *P2X1* deficiency resulted in significantly delayed in vitro cell growth in THP-1, U937 and MV4-11 cells (Fig. S6D–F). An in vivo transplantation assay showed that the recipient mice receiving *P2X1*-knockdown THP-1 cells had reduced leukemia cell frequencies in the peripheral blood (Fig. S6G) and prolonged overall survival (24 or 38 days vs. 15 days, Fig. S6H) compared to control mice.

In the present study, we also found human AML-LICs had the highest expression levels of *P2X1* among non-LICs, total AML cells, normal HSCs, normal-non-HSCs and normal total hematopoietic cells (Fig. 6C). A whole-cell patch-clamp recording assay showed that P2X1-mediated ion flux was elicited upon ATP stimulation and selectively inhibited by PPNDS (Fig. 6D). In addition, *P2X1*-knockdown immunophenotypic human primary CD34^+^ AML-LICs grew much slower in vitro (Fig. 6E–G) and resulted in approximately 50% fewer colony counts or 40% fewer derived total AML cells than the control cells (Fig. 6H–J).

Mechanistically, we noticed that knockdown of *P2X1* in either human primary AML-LICs (Fig. S6I–K) or human AML cell lines (THP-1, U937, MV4-11; Fig. S6L–N) resulted in the significant reduction in the expression levels of *BCAT2, BCAT1, MEIS1, HOXA9, PBX1,* and *PBX3*. Furthermore, we had mutated the phosphorylation sites in human AML cell lines and found that neither S387 A nor T389A rescued the impaired leukemogenic activities of the *P2X1*-knockdown human AML cell lines, such as THP-1 and MV4-11 cells (Fig. S6O, P). These data suggested that P2X1 is important for the leukemogenic activities of both human AML cell lines and primary AML-LICs.

### P2X1 antagonist efficiently inhibits AML cell proliferation

**Fig. 7:**
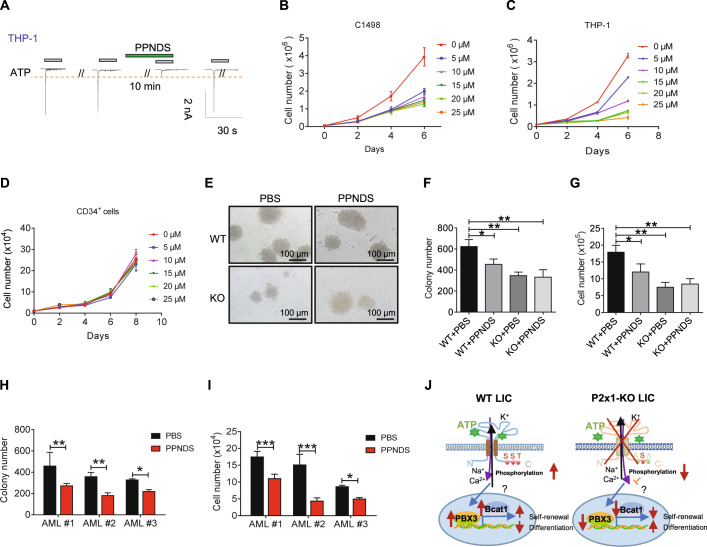
The P2X1 antagonist efficiently suppresses AML cell proliferation. **A** The P2X1-mediated ion influx in THP-1 cells was measured upon sequential treatments with extracellular ATP and the P2X1 antagonist PPNDS by whole-cell patch-clamp recording. Data from three independent experiments. **B**–**D** The indicated doses of PPNDS were used for in vitro treatments of C1498 cells (**B**), THP-1 cells (**C**) and human cord blood CD34^+^ hematopoietic stem progenitor cells (**D**) followed by the determination of total cell counts (*n* = 3). **E** Representative images of colonies derived from WT and *P2x1*-KO murine AML cells treated with PPNDS (5 μM) or PBS. **F**, **G** The colony numbers (**F**) and derived total cell counts (**G**) of WT and *P2x1*-KO murine AML cells were counted after treatment with PPNDS (5 μM) or PBS for 5 days (*n* = 3). **H**, **I** The colony numbers (H) and derived total cell counts (**I**) of patient primary AML cells were counted after treatment with PPNDS (5 μM) or PBS for 7 days (*n* = 3). **J** A working model for the effect of P2X1 on the fate determination of AML-LICs, including self-renewal and differentiation, which is fine-tuned by P2X1 phosphorylation levels at sites of S387 or T389 and its downstream PBX3-BCAT1 pathways (S serine; T threonine; A alanine). Data are presented as the mean ± SD. Two-way ANOVA with Sidak’s multiple comparison test (**F**–**I**) were used for the comparison of statistical significance (*P < 0.05; **P < 0.01; and ***P < 0.001).

To evaluate potential applications of P2X1 blockade in AML treatment, we examined the blocking effect of the P2X1 antagonist, PPNDS, on the ion flux triggered by extracellular ATP. The results showed that PPNDS selectively inhibited ion flux in THP-1 cells (Fig. 7A). Both murine (C1498) and human (THP-1) AML cell lines grew significantly slower than control cells in a dose-dependent manner after PPNDS treatment (Fig. 7B, C). In contrast, PPNDS did not affect the proliferation of normal hematopoietic cells, such as human cord blood CD34^+^ hematopoietic stem progenitor cells (Fig. 7D). Because no suitable P2X1 antagonists were available for in vivo treatment in leukemic mice, we performed an in vitro functional colony formation assay. PPNDS treatment resulted in an approximately 30% decrease in colony numbers and total derived cell counts from murine WT AML cells but not *P2x1*-null AML cells (Fig. 7E–G). Consistently, PPNDS sufficiently suppressed the colony formation of human primary AML cells as indicated by a reduction in colony numbers and their derived cell numbers (Fig. 7H, I), suggesting that PPNDS may provide a novel way to efficiently target AML cells or LICs in the clinic. In summary, ATP-P2X1-mediated signaling was required for the leukemogenic activities of both murine and human LICs. P2X1 phosphorylation levels at specific sites and the downstream PBX3-BCAT1 pathways enhanced AML development (Fig. 7J).

## Discussion

The mechanism by which microenvironmental/niche components contribute to tumorigenesis or leukemogenesis is poorly understood. Extracellular ATP amounts in niches are relatively low (at nanomolar levels) in normal tissues, but they can be increased up to micromolar levels in many pathological states [15, 43]. Although it has been reported that extracellular ATP levels enhance the proliferation of cancer cells [44, 45], some studies have also shown that extracellular ATP inhibits cancer cell growth [46]. These controversial findings may be due to the differential expression of different P2 receptor subtypes [47]. Our previous study revealed that P2X7 enhance leukemia progress via PHGDH-mediated serine metabolic pathways [33]. In the present study, we further showed that P2X1, but not P2X4 or P2X5, promoted leukemogenesis through the PBX3-BCAT1 pathways. The mechanisms by which only P2X1 and P2X7 are involved in AML development, but not P2X4 and P2X5 (Fig. S1H, I), remain unclear, although all four members are highly expressed in AML cells [33, 48].

In the present study, we demonstrated that P2X1 served as an oncogene to enhance LIC self-renewal. However, the roles of P2X1 in other AML types or other leukemia types remain to be further investigated. Interestingly, we showed that P2X1 knockdown in several human primary leukemia cell lines from other FAB-AML types also led to a significant delay in cell proliferation, indicating that P2X1 may exert its oncogenic effects on a variety of cancer types. P2X1 has been reported to enhance mitochondrial activities in T-cell acute lymphoblastic leukemia (T-ALL) cells [49]. Consistently, we also showed that P2X1 augmented BCAT1-mediated BCAA catabolism, which is reported to contribute to enhanced oxidative phosphorylation [50, 51]. These results implicate that metabolic niche factors, such as ATP, may be tightly connected with the intrinsic metabolism of leukemia cells.

P2X1 has been reported to be basally phosphorylated at multiple sites, and it has a conserved intracellular protein kinase C (PKC) motif of TXK/R at the amino terminus [42, 52]. Phosphorylation at the T18P19R20 PKC sites of P2X1 may be required for its physiological functions, and mutation of these proteins leads to an increase in the desensitization rate and a decrease in the peak amplitude of ion flux [52]. However, P2X1 activities may not be directly induced by its phosphorylation level but may be mediated by staurosporine-sensitive phosphorylation of its cofactors [53]. Interestingly, we showed that P2X1 phosphorylation at sites S387 and T389, but not S388, was critical for its effects on enhancing AML development. However, it remains unclear how these two functional phosphorylation sites are regulated. Moreover, the underlying mechanisms related to P2X1 phosphorylation levels in the upregulation of PBX3 expression remain unclear.

Although relapse and drug resistance commonly occur, chemotherapy is still the major treatment for AML in the clinic to date. We have previously shown that inhibition of P2X7 by its specific antagonist (A-740003) efficiently delays AML development [33]. In the present study, we also showed that the P2X1 antagonist (PPNDS) significantly decreased AML cell proliferation. It is possible that combinational treatments targeting both P2X1 and P2X7 may be more potent in blocking AML progression or drug resistance. More efforts are required for screening other antagonists with better performance in AML treatment. In summary, the present study demonstrated that P2X1 enhances LIC self-renewal and suppresses its differentiation via PBX3-BCAT1 signaling without affecting normal hematopoiesis. These results indicated that targeting P2X1 may be an efficient approach for the treatment of AML or other cancers.

## Methods

### Animals

The CD45.1 mice were provided by Dr. Jiang Zhu at Ruijin Hospital, Shanghai, China. The *P2x1*-knockout (KO), *P2x4*-knockout (*P2x4*-KO) and *P2x5*-knockout (*P2x5*-KO) mice in a C57BL/6 background were generated at the Animal Core Facility at the School of Basic Medicine, Shanghai Jiao Tong University School of Medicine. C57BL/6 CD45.2 and NOD-SCID mice were ordered from Shanghai SLAC Laboratory Animal Co. Ltd and maintained at Animal Core Facility at Shanghai Jiao Tong University School of Medicine. All animal experiments were approved and conducted according to the Guidelines for Animal Care at Shanghai Jiao Tong University School of Medicine. All the other information related to the Methods is in the Supplementary Data file.

## Supplementary information

SUPPLEMENTARY MATERIAL

SUPPLEMENTARY MATERIAL

## Data Availability

All data generated or analyzed during this study are included in this published article and its supplementary information files. Further inquiries can be directed to the corresponding authors.
