# Supplementary material for: P2X1 enhances leukemogenesis through PBX3-BCAT1 pathways
Source: Leukemia. 2022 Nov 23;37(2):265–75. doi: 10.1038/s41375-022-01759-y (PMC9898031; doi:10.1038/s41375-022-01759-y)
Supplement: Supplementary file 1 — SUPPLEMENTARY MATERIAL [file 41375_2022_1759_MOESM1_ESM.pdf]

Supplementary materials

Figure S1

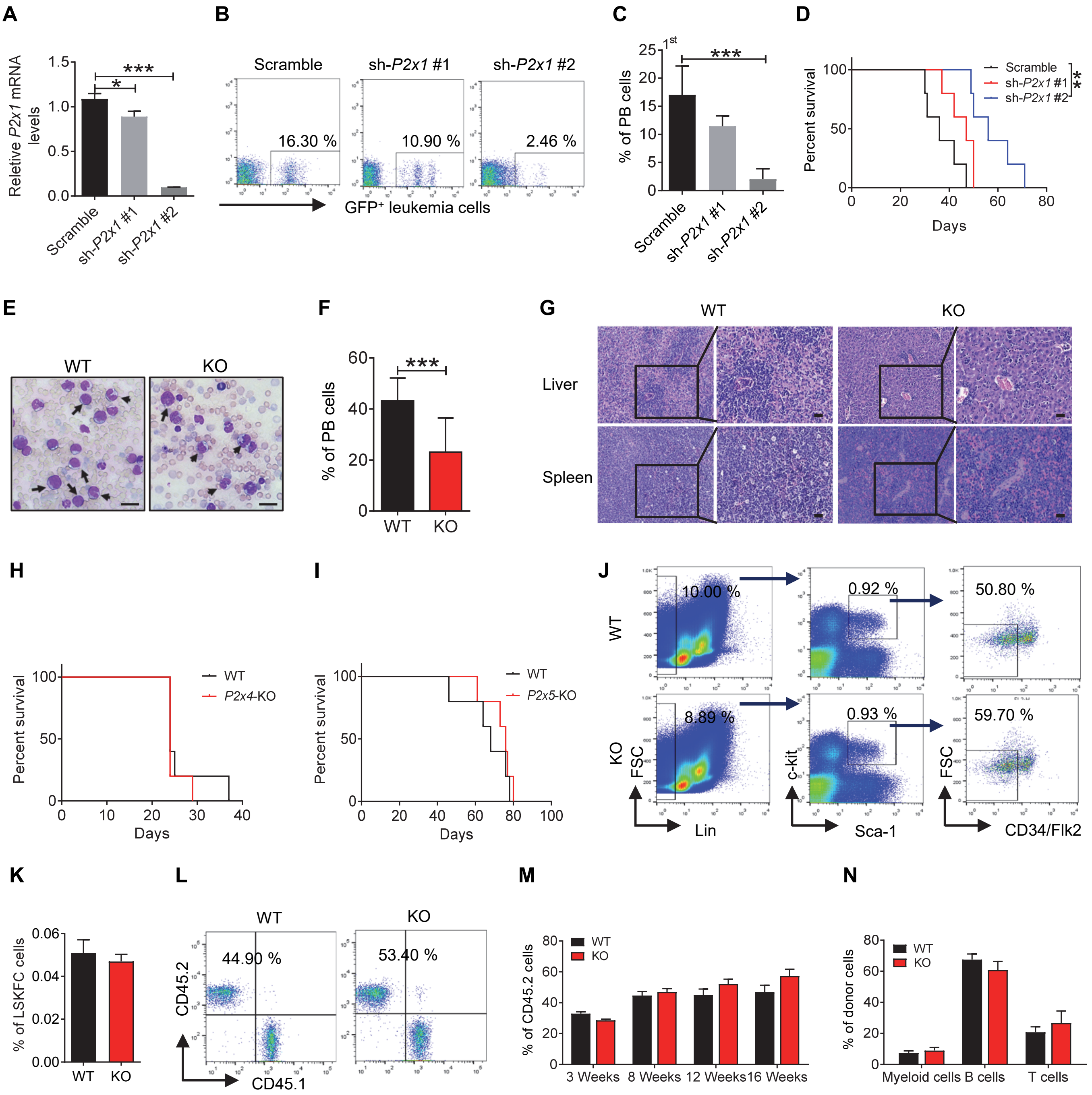

Figure S1 (related to Figure 1). P2X1 is highly enriched in LICs and required for leukemogenesis. (A) The knockdown efficiencies of shRNAs (sh-*P2x1* #1 and #2) targeting murine *P2x1* were determined by qRT–PCR (n=3). (B) Representative flow cytometric analysis of GFP^{+} AML cells in the peripheral blood of recipient mice receiving transplants of *P2x1*-knockdown GFP^{+}MLL-$AF9^{+}$ BM cells or scrambled controls upon primary transplantation. (C) Quantification of the frequencies of AML cells shown in B (n=5). (D) The overall survival of the recipient mice transplanted with P2x1-knockdown GFP^{+}MLL-$AF9^{+}$ BM cells or scrambled controls upon primary transplantation (n=5). (E) Representative images of Giemsa-Wright staining in WT and *P2x1*-KO AML cells from the peripheral blood of recipient mice upon primary transplantation. (F) Quantification of the frequencies of blast cells shown in E (n=3-5). (G) Representative images of hematoxylin-eosin staining in the livers and spleens of recipients transplanted with WT and *P2x1*-KO AML cells upon primary transplantation. (H) The overall survival of recipient mice transplanted with WT or *P2x4*-KO MLL-$AF9^{+}$ BM cells upon primary transplantation (n=5). (I) The overall survival of recipient mice transplanted with WT or *P2x5*-KO MLL-$AF9^{+}$ BM cells upon primary transplantation (n=5). (J) Representative flow cytometric analysis of Lin^{-}Scal-1^{+}c-$Kit^{+}CD34^{-}Flk2^{-}$ HSCs in the BM of WT and *P2x1*-KO mice. (K) Quantification of the frequencies of HSCs shown in J (n=5). (L) Representative flow cytometric analysis of donor cells (CD45.2) in the peripheral blood of recipient mice 16 weeks after BM competitive transplantation. (M) The frequencies of WT and *P2x1*-KO donor cells were evaluated 3, 8, 12 and 16 weeks after transplantation. (N) Multilineages of donor cells in recipient mice transplanted with WT and *P2x1*-KO BM cells 16 weeks post transplantation (n=5). Scale bar, 20 μm. Data are presented as the mean ± SD. One-way ANOVA with Tukey's multiple comparison test (A and C), log-rank test (D, H and I),

Student's two-tailed unpaired *t* test (F and K) and two-way ANOVA with Sidak's multiple comparison test (M and N) were used for the comparison of statistical significance (*, $P<0.05$; **, $P<0.01$; and ***, $P<0.001$).

Figure S2

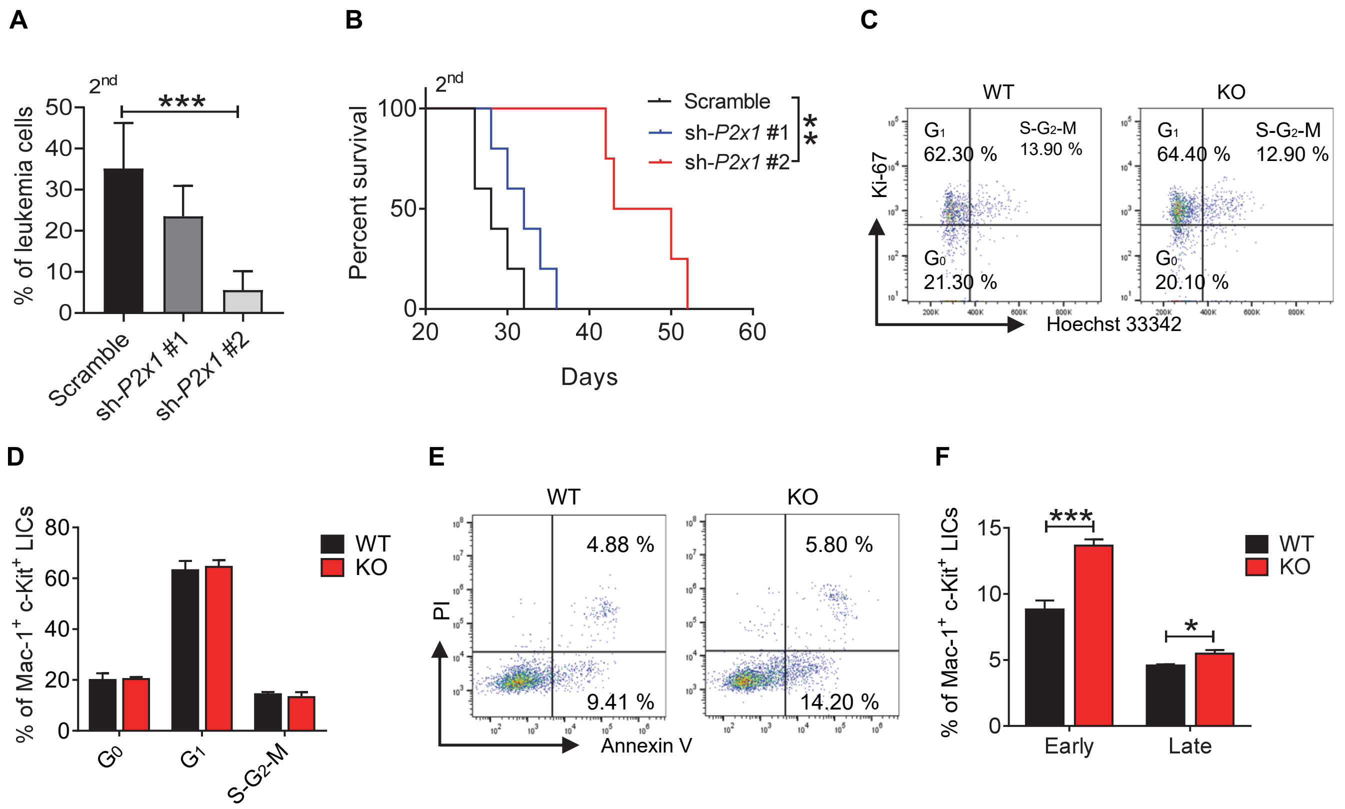

A
2nd
***
% of leukemia cells
50
40
30
20
10
0
Scramble
sh-P2x1 #1
sh-P2x1 #2
B
2nd
Percent survival
100
50
0
20
30
40
50
60
Days
Scramble
sh-P2x1 #1
sh-P2x1 #2
**
C
WT
KO
G1
62.30 %
S-G2-M
13.90 %
G0
21.30 %
G1
64.40 %
S-G2-M
12.90 %
G0
20.10 %
Ki-67
Hoechst 33342
D
% of Mac-1+ c-Kit+ LICs
80
60
40
20
0
WT
KO
G0
G1
S-G2-M
E
WT
KO
4.88 %
9.41 %
5.80 %
14.20 %
PI
Annexin V
F
% of Mac-1+ c-Kit+ LICs
15
10
5
0
***
*
WT
KO
Early
Late

Figure S2 (related to Figure 2). P2X1 promotes self-renewal and inhibits the differentiation of LICs. (A) Quantification of the frequencies of GFP$^+$ AML cells in the peripheral blood of recipient mice that received transplants of *P2x1*-knockdown MLL-AF9$^+$ BM cells or scrambled controls upon secondary transplantation (n=5). (B) The overall survival of the recipient mice transplanted with *P2x1*-knockdown MLL-AF9$^+$ BM cells or scrambled controls upon secondary transplantation (n=5). (C) Cell cycle status was determined in WT and *P2x1*-KO Mac-1$^+$c-Kit$^+$ LICs of the recipients. (D) Quantification data of the phases of cell cycle in panel C. One representative out of two independent experiments with n=3 mice per group is shown. (E) Representative flow cytometric analysis of apoptosis of WT or *P2x1*-KO Mac-1$^+$c-Kit$^+$ LICs. (F) Quantification of data in panel E. One representative out of two independent experiments with n=3 mice per group is shown. Data are presented as the mean ± SD. One-way ANOVA with Tukey's multiple comparison test (A), log-rank test (B) and two-way ANOVA with Sidak's multiple comparison test (D, F) were used for the comparison of statistical significance. (*, P<0.05**, P<0.01; and ***, P<0.001).

Figure S3

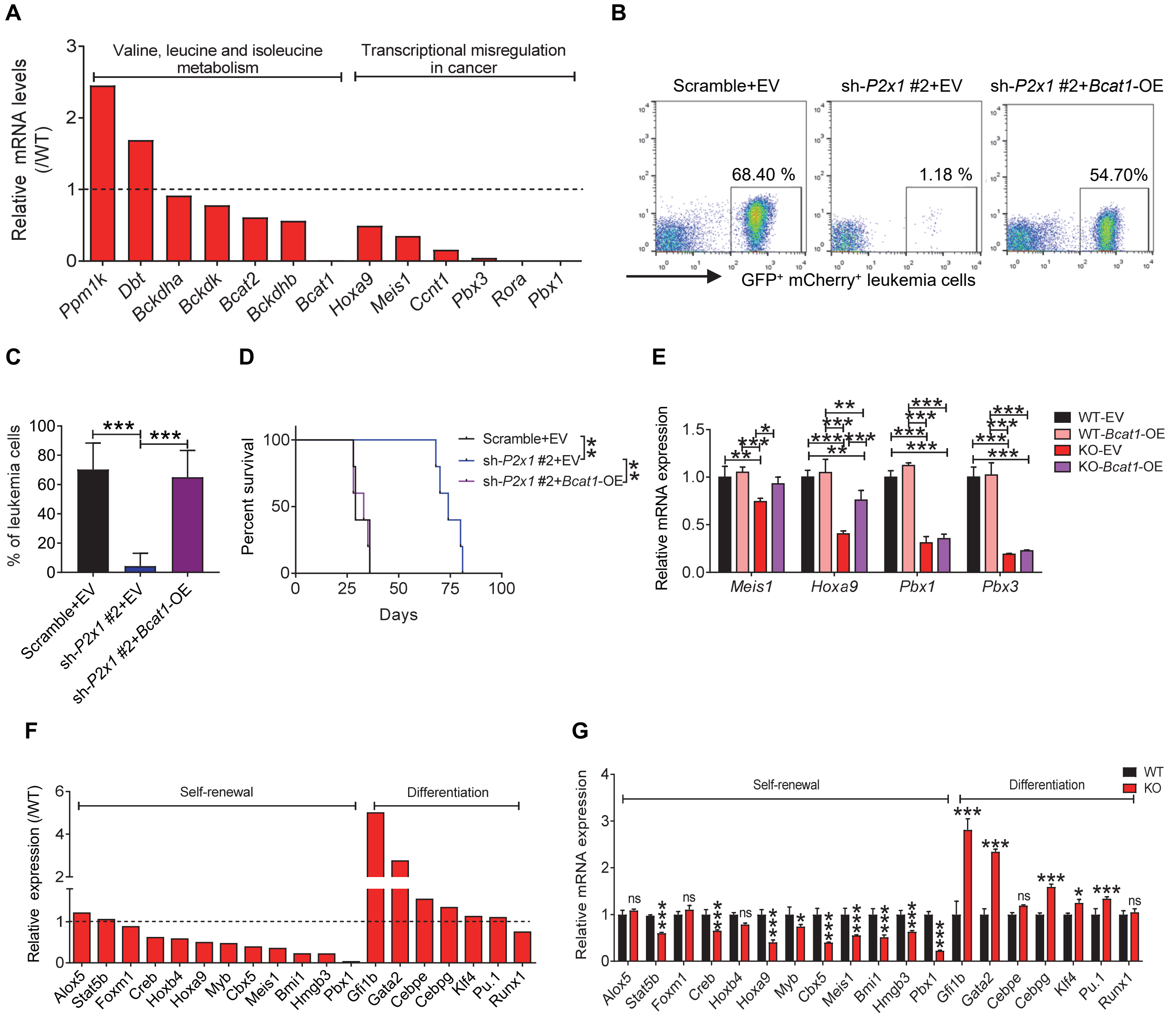

A
Valine, leucine and isoleucine metabolism
Transcriptional misregulation in cancer
Relative mRNA levels (/WT)
Ppm1k
Dbt
Bckdha
Bckdk
Bcat2
Bckdhb
Bcat1
Hoxa9
Meis1
Ccnt1
Pbx3
Rora
Pbx1
B
Scramble+EV
sh-P2x1 #2+EV
sh-P2x1 #2+Bcat1-OE
68.40 %
1.18 %
54.70%
GFP+ mCherry+ leukemia cells
C
% of leukemia cells
Scramble+EV
sh-P2x1 #2+EV
sh-P2x1 #2+Bcat1-OE
D
Percent survival
Days
Scramble+EV
sh-P2x1 #2+EV
sh-P2x1 #2+Bcat1-OE
E
Relative mRNA expression
WT-EV
WT-Bcat1-OE
KO-EV
KO-Bcat1-OE
Meis1
Hoxa9
Pbx1
Pbx3
F
Relative expression (/WT)
Self-renewal
Differentiation
Alox5
Stat5b
Foxm1
Creb
Hoxb4
Hoxa9
Myb
Cbx5
Meis1
Bmi1
Hmgb3
Pbx1
Gfi1b
Gata2
Cebpe
Cebpg
Klf4
Pu.1
Runx1
G
Relative mRNA expression
Self-renewal
Differentiation
WT
KO

Figure S3 (related to Figure 3). P2x1 regulates LIC activities via BCAT1-mediated branched chain amino acid metabolism. (A) Candidate genes related to valine, leucine and isoleucine metabolism were analyzed using RNA-sequencing data from WT and *P2x1*-KO Mac-1^{+}c-Kit^{+} LICs. (B) Representative flow cytometric analysis of leukemia cells (GFP^{+}mCherry^{+}, which were the tags for the pLKO.1-GFP plasmid and MSCV-mCherry overexpression plasmid) in the peripheral blood of recipient mice transplanted with *P2x1*-knockdown leukemia cells, *Bcat1*-overexpressing *P2x1*-knockdown leukemia cells or scrambled control cells. (C) Quantification of the data shown in B (n=5). (D) The overall survival was compared among the mice transplanted with *P2x1*-knockdown, *Bcat1*-overexpressing *P2x1*-knockdown or scrambled control cells (n=5). (E) The mRNA levels of *Meis1, Hoxa9, Pbx1* and *Pbx3* in Mac-1^{+}c-Kit^{+} LICs from the recipient mice transplanted with WT, *P2x1*-KO, *Bcat1*-overexpressing WT or *P2x1*-KO AML cells were measured by qRT-PCR (n=3). (F) Candidate genes related to self-renewal and differentiation were analyzed using RNA-sequencing data from WT and *P2x1*-KO Mac-1^{+}c-Kit^{+} LICs. (G) Potential candidates related to self-renewal and differentiation were examined in WT and *P2x1*-KO Mac-1^{+}c-Kit^{+} LICs by qRT-PCR (n=3). Data are presented as the mean ± SD. One-way ANOVA with Tukey's multiple comparison test (C), log-rank test (D), and two-way ANOVA with Sidak's multiple comparison test (E, G) were used for the comparison of statistical significance (*, $P<0.05$; **, $P<0.01$; and ***, $P<0.001$).

Figure S4

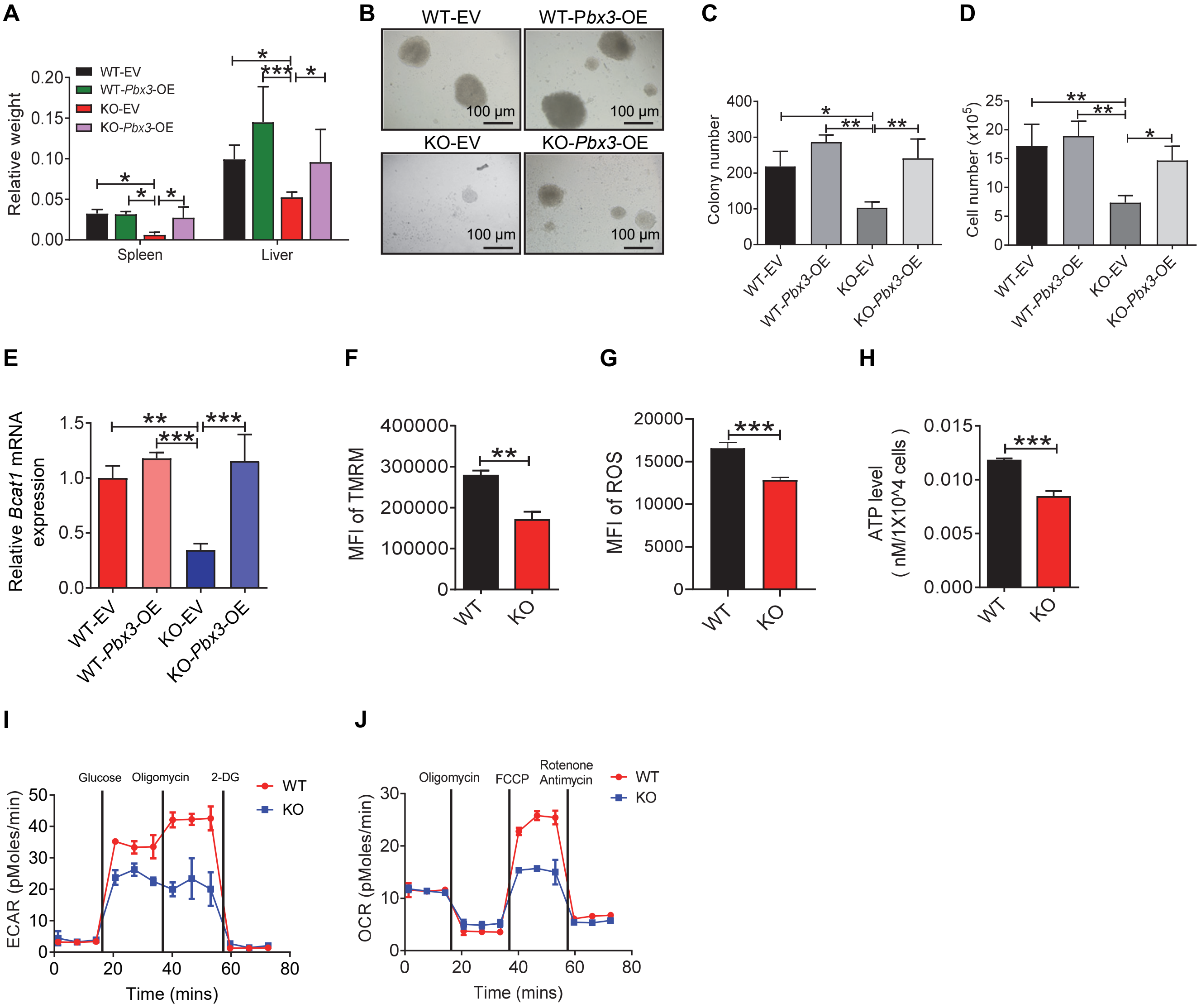

A
B
C
D
E
F
G
H
I
J
WT-EV
WT-Pbx3-OE
KO-EV
KO-Pbx3-OE
Relative weight
Spleen
Liver
100 μm
Colony number
Cell number (x10^5)
Relative Bcat1 mRNA expression
MFI of TMRM
MFI of ROS
ATP level (nM/1X10^4 cells)
WT
KO
Glucose
Oligomycin
2-DG
ECAR (pMoles/min)
Time (mins)
FCCP
Rotenone Antimycin
OCR (pMoles/min)

Figure S4 (related to Figure 4). PBX3 transactivates BCAT1 expression to sustain LIC activities. (A) Quantification data of the spleen and liver sizes of recipient mice transplanted with WT, *P2x1*-KO, *Pbx3*-overexpressing WT or *P2x1*-KO leukemia cells (n=3). (B) Representative images of colonies derived from WT, *P2x1*-KO, *Pbx3*-overexpressing WT or *P2x1*-KO YFP$^+$mCherry$^+$ AML cells. (C and D) Quantification data of colony numbers (C) and derived total cell counts (D) shown in B (n=3). (E) The *Bcat1* mRNA levels in BM cells from recipient mice transplanted with WT, *P2x1*-KO, *Pbx3*-overexpressing WT or *P2x1*-KO AML cells were measured by qRT–PCR (n=3). (F) The mean fluorescence intensities (MFI) of TMRM in WT, *P2x1*-KO Mac-1$^+$c-Kit$^+$ LICs. One representative out of three independent experiments with n=3 mice per group is shown. (G) The levels of ROS in WT and *P2x1*-KO Mac-1$^+$c-Kit$^+$ LICs were determined by flow cytometry. One representative out of three independent experiments with n=3 mice per group is shown. (H) The levels of ATP of WT and *P2x1*-KO Mac-1$^+$c-Kit$^+$ LICs were measured by a bioluminescence assay. (I-J) Extracellular acidification rates (ECAR, I) and oxygen consumption rate (OCR, J) were measured in WT and *P2x1*-KO Mac-1$^+$c-Kit$^+$ LICs using a Seahorse XF96 analyzer (n=3). 2-DG, 2-deoxyglucose. mpH, mili potential of hydrogen. Data are presented as the mean ± SD. Two-way ANOVA with Sidak's multiple comparison test (A) one-way ANOVA with Tukey's multiple comparison test (C-E) and Student's two-tailed unpaired t test (F-H) were used for the comparison of statistical significance (*, $P<0.05$; **, $P<0.01$; and ***, $P<0.001$).

Figure S5

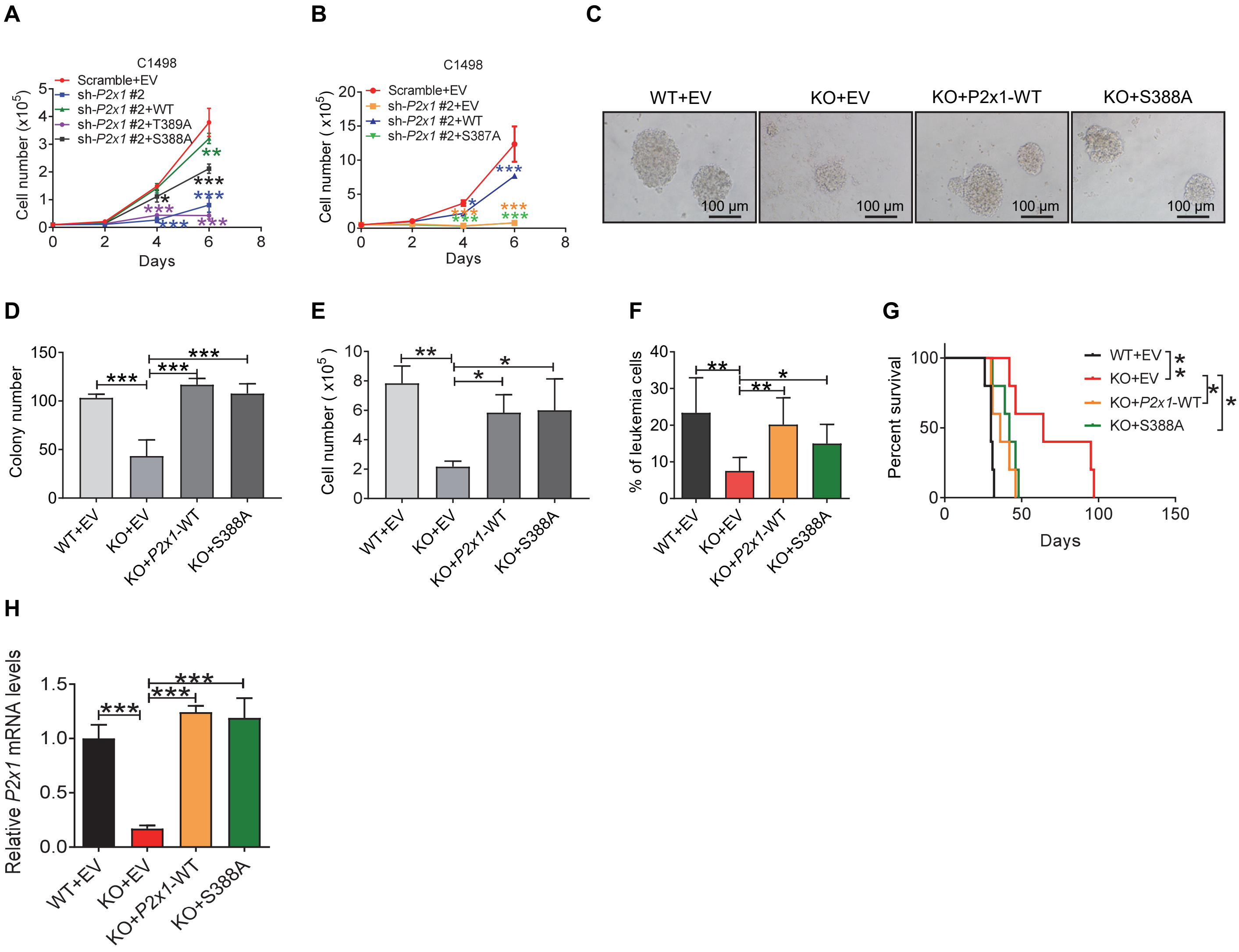

A
C1498
Scramble+EV
sh-P2x1 #2
sh-P2x1 #2+WT
sh-P2x1 #2+T389A
sh-P2x1 #2+S388A
Cell number (x10^5)
Days
B
C1498
Scramble+EV
sh-P2x1 #2+EV
sh-P2x1 #2+WT
sh-P2x1 #2+S387A
Cell number (x10^5)
Days
C
WT+EV
KO+EV
KO+P2x1-WT
KO+S388A
100 µm
D
Colony number
E
Cell number (x10^5)
F
% of leukemia cells
G
Percent survival
Days
H
Relative P2x1 mRNA levels
WT+EV
KO+EV
KO+P2x1-WT
KO+S388A

Figure S5 (related to Figure 5). P2X1 phosphorylation sites at S387 and T389 are critical for leukemogenic activities. (A) The scrambled and *P2x1*-knockdown C1498 cells (by a shRNA targeting murine *P2x1*, sh-*P2x1* #2) were infected with empty vector (EV), WT or mutant *P2x1* (S388A or T389A), and cell numbers were counted at the indicated days (n=3). (B) The scrambled and *P2x1*-knockdown C1498 cells (sh-*P2x1* #2) were infected with empty vector (EV), WT or mutant *P2x1* (S387A), and cell numbers were counted at the indicated days (n=3). (C) Representative images of colonies derived from WT and *P2x1*-KO AML cells ectopically expressing empty vector (EV), WT or mutant *P2x1* (S388A). (D and E) Quantification of the colony numbers (D) and derived total cell counts (E) shown in C (n=3). (F) Quantification of the frequencies of AML cells in the peripheral blood of recipient mice receiving WT or *P2x1*-KO AML cells ectopically expressing empty vector (EV), WT or mutant *P2x1* (S388A) (n=5). (G) The overall survival was examined in recipient mice transplanted with WT or *P2x1*-KO AML cells ectopically expressing empty vector (EV), WT or mutant *P2x1* (S388A) (n=5). (H) The *P2x1* mRNA levels in BM cells of recipient mice transplanted with WT or *P2x1*-KO AML cells ectopically expressing empty vector (EV), WT or mutant *P2x1* (S388A) were measured by qRT–PCR (n=3). Data are presented as the mean ± SD. Two-way ANOVA with Sidak's multiple comparison test (A and B), one-way ANOVA with Tukey's multiple comparison test (D, E, F and H) and log-rank test (G) were used for the comparison of statistical significance (*, $P<0.05$; **, $P<0.01$; and ***, $P<0.001$).

Figure S6

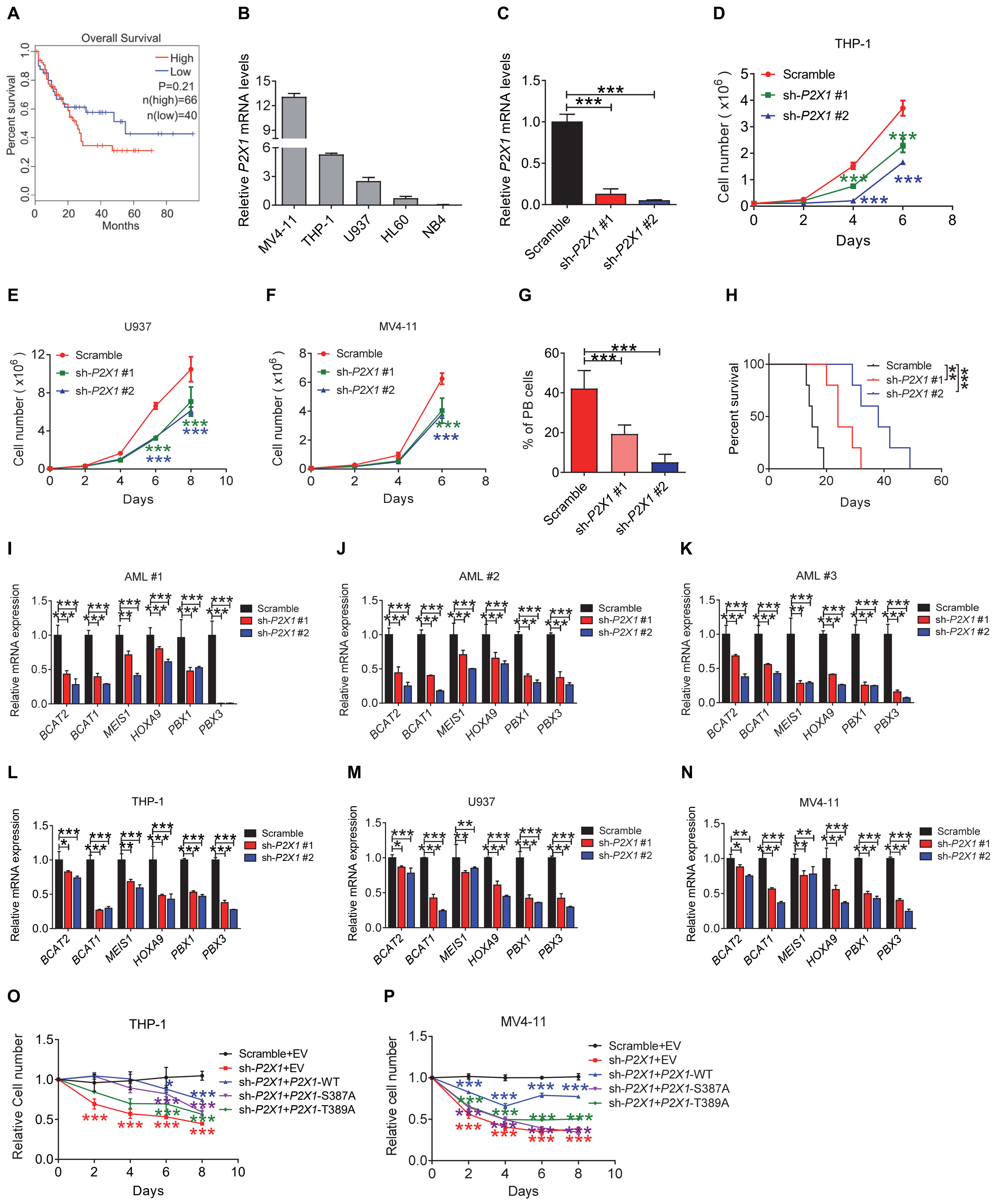

A
Overall Survival
High
Low
P=0.21
n(high)=66
n(low)=40
Percent survival
Months
B
Reletive P2X1 mRNA levels
MV4-11
THP-1
U937
HL60
NB4
C
Reletive P2X1 mRNA levels
Scramble
sh-P2X1 #1
sh-P2X1 #2
D
THP-1
Scramble
sh-P2X1 #1
sh-P2X1 #2
Cell number (x10^6)
Days
E
U937
F
MV4-11
G
% of PB cells
H
Percent survival
Days
I
AML #1
J
AML #2
K
AML #3
Relative mRNA expression
BCAT2
BCAT1
MEIS1
HOXA9
PBX1
PBX3
L
THP-1
M
U937
N
MV4-11
O
THP-1
Relative Cell number
Scramble+EV
sh-P2X1+EV
sh-P2X1+P2X1-WT
sh-P2X1+P2X1-S387A
sh-P2X1+P2X1-T389A
P
MV4-11
Relative cell number

Figure S6 (related to Figure 6). P2X1 is required for the proliferation of human AML cells. (A) Relationship between *P2X1* mRNA levels and overall survival in AML patients from TCGA databases (high, n=66; low, n=40). (B) The mRNA levels of *P2X1* in MV4-11, THP-1, U937, HL60 and NB4 cells were measured by qRT–PCR (n=3). (C) The knockdown efficiencies of *P2X1* shRNAs specifically targeting human *P2X1* (sh-*P2X1* #1 and #2) were measured in THP-1 cells by qRT–PCR (n=3). (D-F) The numbers of THP-1, U937 and MV4-11 cells were counted at the indicated days after infection with P2X1 shRNAs (sh-*P2X1* #1 and #2) or a scrambled shRNA (n=3). (G) Quantification of the frequencies of AML cells in the peripheral blood of recipient mice that received transplants of *P2X1*-knockdown THP-1 cells or scrambled cells (n=5). (H) The overall survival was evaluated in recipient mice transplanted with *P2X1*-knockdown (sh-*P2X1* #1 and #2) THP-1 cells or control cells (n=5). (I-K) The mRNA levels of *BCAT2, BCAT1, MEIS1, HOXA9, PBX1* and *PBX3* in human primary AML cells (AML#1-#3) upon the knockdown of *P2X1* by shRNAs (sh-*P2X1* #1 and #2) and the scrambled one were examined by qRT-PCR. (L-N) The mRNA levels of *BCAT2, BCAT1, MEIS1, HOXA9, PBX1* and *PBX3* in THP-1 (L), U937 (M) and MV4-11 cells (N) upon the knockdown of *P2X1* by shRNAs (sh-*P2X1* #1 and #2) and the scrambled one were examined by qRT-PCR (n=3). O-P) The scrambled and *P2X1*-knockdown THP-1 (O) and MV4-11 (P) cells (by a shRNA targeting human *P2X1*, sh-*P2X1* #2) were infected with empty vector (EV), WT or mutant *P2X1* (S387A or T389A) plasmids. Cell numbers were counted at the indicated days (n=3). Data are presented as the mean ± SD. One-way ANOVA with Tukey's multiple comparison test (C and G), two-way ANOVA with Sidak's multiple comparison test (D-F and I-P) and log-rank test (H) were used for the comparison of statistical significance (*, $P<0.05$, **, $P<0.01$; and ***, $P<0.001$).

Supplemental Methods and Materials

AML cell lines

Several murine or human AML cell lines, including C1498 (murine, AML), THP-1 (human, M5), MV4-11 (human, M5), U937 (human, M5), HL60 (human, M3) and NB4 (human, M3) were cultured in RPMI 1640 medium (Hyclone, UT, USA) supplemented with 10% FBS (ExCell, China). All the cell lines were ordered from ATCC.

Antibodies and Reagents

Antibodies: human hematopoietic lineage eFluor 450 Cocktail (eBioscience, CA, USA, #85-22-7775-72), anti-human CD34-FITC (eBioscience, #11-0349), anti-human CD38-PE-Cy7 (eBioscience, #85-25-0388-41), anti-human CD90-PerCP-Cy5.5 (eBioscience, #85-12-0909-42), anti-human CD45RA-PE (eBioscience, #85-12-0458-42), biotin-conjugated mouse lineage depletion cocktails (BD Pharmingen, NJ, USA #2160546), streptavidin PerCP-Cyanine5.5 (eBioscience, #45-4317-82), anti-mouse Sca-1-FITC (eBioscience, #130-102-831), anti-mouse Sca-1-PECy7 (eBioscience, #25-5981-82), anti-mouse c-Kit-APC (eBioscience, #17-1171-82), anti-mouse CD135-PE (eBioscience, #12-1351-82), anti-mouse CD34 eFluor450 (eBioscience, #48-0341-82), anti-mouse CD34-PE (eBioscience, #12-0349-42), anti-mouse CD16/CD32-PE (eBioscience, #85-12-0161-81), anti-mouse CD16/32-eFluor450 (eBioscience, #48-0161-82), anti-mouse CD127-Biotin (eBioscience, #13-1271-82), anti-mouse Gr-1-Biotin (eBioscience, #13-5931-85), anti-mouse Ter-119-Biotin (eBioscience, #13-5921-85), anti-mouse CD45.2-FITC (eBioscience, #11-0454-82), anti-mouse CD45.1-PE (eBioscience, #12-0453-82), anti-mouse CD3e-APC (eBioscience, #17-0031-82), anti-mouse B220-PE (eBioscience, #12-0452-85), anti-mouse Mac-1-APC (eBioscience, #17-0112-82), anti-mouse Gr-1-PE (eBioscience, #12-5931-82), Ki-67

Set (BD Pharmingen, #556027), AnnexinV Apoptosis Detection Kit (BD Pharmingen, #559763), EasySep™ human CD34 Positive Selection Kit (StemCell Technologies, Canada, #18056), TMRM (Sigma-Aldrich, USA, #T5428), Fluorometric Intracellular Ros deep red Kit (Sigma-Aldrich, #MAK142), β-actin (Calbiochem, Germany, #CP01), P2X1 (Origene, China, #TA329003).

Reagents: Poly-D-lysine hydrobromide (Sigma-Aldrich, #P7886), ChIP Assay Kit (Beyotime, China, #P2078), XF Cell Mito Stress Test Kit (Agilent, China, #103015-100), XF Glycolysis Stress Test Kit (Agilent, #103020-100), ATP Bioluminescence Assay Kit HS II (Roche, China, #11699709001), PPNDS (Tocris, UK, #1309/10).

Establishment of AML model and BM competitive transplantation

To establish a murine AML model, an MSCV-MLL-AF9-IRES-YFP-encoding plasmid and a pCL-ECO packaging plasmid (2:1) were co-transfected into 293T cells to produce retroviruses. Retroviruses were used for the infection of isolated wild-type (WT) and *P2x1* KO Lin^- fetal liver cells by spinning infection in presence of 4 μg/mL polybrene. Infected cells ($1\text{-}3\times10^5$) were transplanted into lethally irradiated C57BL/6 WT recipient mice by retroorbital injection, followed by the analysis of the leukemia cell frequencies in the peripheral blood and overall survival of leukemic mice. Serial transplantations were performed with the same number of purified YFP^+ BM AML cells. The limiting dilution assay was performed with the indicated YFP^+ BM leukemia cells (100, 300 and 1000), followed by calculation of functional LIC frequencies (Table S1) according to the overall survival of recipients using the L-Calc software (Stemcell Technologies). In some cases, the shRNA plasmids (pLKO.1-IRES-GFP) specifically targeting murine *P2x1* or human *P2X1* were co-transfected with pSPAX2 and pMD2G packaging plasmids (4:3:1) into 293T cells. Lentiviruses were used for the subsequent

infection with MLL-AF9-YFP$^+$ BM leukemia cells, THP-1/U937/MV4-11 cells (three human AML cell lines, ATCC) or patient primary AML cells and subjected to the analysis of *in vitro* cell proliferation. In some cases, FACS-purified murine YFP$^+$ BM leukemia cells (1×10^4) or human GFP$^+$ THP-1 cells (2×10^6) were injected into lethally irradiated C57BL/6 mice or sublethally irradiated NOD-SCID mice, respectively, followed by the evaluation of leukemia development.

For rescue experiments, retroviral plasmid MSCV-HA-*Bcat1*-mCherry, MSCV-HA-*Pbx3*-mCherry, MSCV-HA-*P2x1*-WT-mCherry or MSCV-HA-*P2x1*- mutant-mCherry plasmid was mixed with pCL-ECO packaging plasmid at a ratio of 2:1 and transfected into 293T cells. Virus-containing supernatant was collected for the infection with WT and *P2x1*-KO BM bulk leukemia cells, followed by transplantation into recipient mice.

For the competitive reconstitution analysis, a total number of 3×10^5 WT or *P2x1*-KO CD45.2 donor BM cells were mixed with 3×10^5 CD45.1 competitor BM cells and transplanted into lethally irradiated CD45.1 recipient mice by retroorbital injection. The repopulated donor cells in the peripheral blood were analyzed at 3, 8, 12 and 16 weeks after transplantation. Multilineages of hematologic cells in the peripheral blood were also determined 16 weeks after transplantation.

RNA-sequencing and quantitative RT-PCR

WT and *P2x1*-KO Mac-1$^+$c-Kit$^+$ LICs from 3 leukemic mice were sorted by flow cytometry, pooled for the extraction of total RNA and subjected to RNA-sequencing at Shanghai Lie Bing Biomedical Technology Co., Ltd. A Gene Ontology enrichment analysis was performed by a Bioconductor package top GO. The enrichment analysis of KEGG pathways was conducted by a Bioconductor package GSEA Base (http://www.genome.jp/Kegg/). We have deposited RNA sequencing data in the GEO

repository (accession#: GSE200724). Candidate genes were further validated with Mac-1^{+}c-Kit^{+} LICs from the recipient mice transplanted with WT, *P2x1*-null, *Bcat1* or *Pbx3* overexpressing WT or *P2x1*-null AML cells, human primary AML-LICs (CD34^{+} cells) or human AML cell lines by qRT-PCR. Murine *P2x1* and human *P2X1* mRNA levels were also measured in murine Mac-1^{+}c-Kit^{+} LICs, Lin^{-}CD127^{-}Sca-1^{-}c-Kit^{+}CD16/32^{+} CD34^{+} L-GMP cells, bulk leukemia cells, Lin^{-} Sca-1^{+}c-Kit^{+}CD34^{-}Flk2^{-} HSCs, human AML cell lines, human Lin^{-}CD34^{+}CD38^{-}CD90^{+}CD45RA^{-} HSCs, human primary CD34^{+} LICs, human normal cord blood cells (Normal-total), normal CD34^{-} cells (Normal-non-HSCs), human primary AML cells (AML-total) or CD34^{-} cells (AML-non-LICs). Briefly, first-strand cDNA was reversely transcribed using AMV reverse transcriptase (Takara, Japan, #2630A). PCR reactions were performed according to the manufacturer's protocol with the Applied Biosystems 7900HT. mRNA levels were normalized to β-actin RNA transcript levels. Primer sequences are shown in Table S2.

Flow cytometry

The WT or *P2x1*-KO/*P2x1*-knockdown immunophenotypic Mac-1^{+}c-Kit^{+} LICs, myeloid or lymphoid lineages were stained with monoclonal antibodies (eBioscience) of anti-Mac-1-APC (or PE), anti-Gr-1-PE, anti-CD3-APC, anti-B220-PE and anti-c-Kit-PE (or APC). Murine Lin^{-}CD127^{-}Sca-1^{-}c-Kit^{+}CD16/32^{+}CD34^{+} L-GMP cells were stained with the biotinylated antibodies of anti-CD127, anti-Gr-1, anti-B220, anti-CD3, anti-CD8 and anti-Ter119, followed by staining with the antibodies of streptavidin-PE/Cy5.5, Sca-1-PE/Cy7, c-Kit-APC, CD16/32-eflour450 and CD34-PE (eBioscience). The repopulation and its multilineages were evaluated in WT and *P2x1*-KO donor total BM cells with antibodies of anti-CD45.1-PE, anti-CD45.2-APC, anti-Mac-1-APC,

anti-Gr-1-PE, anti-CD3-APC and anti-B220-PE. In some cases, $Lin^{-}Sca\text{-}1^{+}c\text{-}Kit^{+}Flk2^{-}CD34^{-}$ LT-HSCs and $Lin^{-}Sca\text{-}1^{+}c\text{-}Kit^{+}Flk2^{-}CD34^{+}$ ST-HSCs, were FACS-purified by staining with a biotinylated lineage cocktail (anti-CD3, anti-CD5, anti-B220, anti-Mac-1, anti-Gr-1, anti-Ter119; BD Pharmingen) followed by streptavidin-PE/Cy5.5, anti-Sca-1-FITC, anti-c-Kit-APC, anti-Flk2-PE, anti-CD34-eFluor450 (eBioscience). Cell cycle status of LICs was determined by Ki-67/Hoechst 33342 staining (BD Pharmingen). For the analysis of the apoptotic status, LICs were stained with anti-annexin V and PI (BD Pharmingen) according to the manufacturer's instructions.

Western blot

WT and *P2x1*-KO BM cell lysates were electrophoresed on 10% SDS polyacrylamide gels and transferred onto nitrocellulose membranes (Millipore, Germany). The membranes were blocked with 5% nonfat milk and incubated with primary antibodies of P2X1 (Origene, #TA329003), followed by incubation with the HRP-conjugated secondary antibodies.

Whole-cell patch-clamp electrophysiological recording

Whole-cell patch-clamp recordings were performed using Axon 200B (Axon Instruments, Foster City, CA, USA) with a voltage clamp. Recording electrodes were filled with a pipette solution. Patch electrodes (3-5 MΩ) were filled with intracellular solution containing 30 mM NaCl, 120 mM KCl, 1 mM $MgCl_2$, 0.5 mM $CaCl_2$, 5 mM EGTA and 10 mM HEPES (pH 7.2). Indicated cells were incubated in a standard extracellular solution with 150 mM NaCl, 10 mM glucose, 5 mM KCl, 1 mM $MgCl_2$, 2 mM $CaCl_2$ and 10 mM HEPES (pH 7.4). $CaCl_2$ and $MgCl_2$ were substituted with 5 mM EGTA in a Ca^{2+}-free solution. Membrane current signals (ion flux) were amplified

by using Axon 200B. Data were sampled at 10 kHz and filtered at 2 kHz. Murine Mac-1^+c-Kit$^+$ LICs, human AML cell line (THP-1) or human Lin$^-$CD34$^+$CD38$^-$CD90$^-$ CD45RA$^+$ primary LICs were seeded on the glass coverslips pretreated with poly-D-lysine (100 μg/mL, Sigma) before electrophysiological recording. The P2X1 specific antagonist, PPNDS, was used for the inhibition of ion flux after ATP stimulation. Data were analyzed using a Digi data 1440 interface and a computer with the Clampex and Clampfit 10.0 software (Molecular Devices). All currents were sampled and analyzed.

Luciferase reporter assay

A luciferase reporter vector pGL4.27 containing a *Bcat1* promoter was constructed to identify the transcriptional activation of *Bcat1* by PBX3. The indicated doses of the MSCV-HA-*Pbx3*-mCherry (or negative control vector) plasmid and the pGL4.27-*Bcat1* promoter vector were co-transfected into 293T cells. Twenty-four hours after transfection, luciferase activities were measured using a luciferase reporting system (GloMax® Multi Instrument). The luciferase measurement is defined as the ratio of firefly luciferase units to Renilla luciferase units.

Chromatin immunoprecipitation (ChIP) assay

ChIP analysis was performed using a ChIP Assay Kit (Beyotime, # P2078). 293T cells were co-transfected with MSCV-HA-*Pbx3*-mCherry and pGL4.27-*Bcat1* promoter plasmids, crosslinked with 1% formaldehyde (Sigma) at 37°C for 10 min, followed by incubation with an anti-HA tag antibody (Abcam, UK, #ab1424) or a rabbit control IgG antibody (CST, MA, USA) at 4°C overnight. For sample input, 1% of ultrasound pre-cleared DNA was purified simultaneously with precipitated immune complex. The ChIP samples were purified by gel and PCR-clean up Kit (Nucleospin, Germany,

#2108/005). The PBX3 binding sequence was amplified by semi-quantitative PCR using *Bcat1* promoter region specific primers as listed in Table S2.

***In vitro* colony formation unit assay and cell proliferation analysis**

Three thousand murine BM AML cells were seeded in a methylcellulose medium (Stem Cell Technologies, #M3534) according to the manufacturer's information. The numbers of colonies and derived cell counts were determined 5-8 days after plating. The same numbers of AML cells from the primary plating were subjected to the secondary plating. In another case, 5 μM of P2X1 antagonist PPNDS (TOCRIS) was added to the methylcellulose medium and colony numbers were determined. For the colony formation assay with human primary AML cells, *P2X1*-knockdown (sh-*P2X1* #1 and #2) and scrambled AML cells were seeded into a methylcellulose medium (Stemcell Technologies, #H4436) according to the manufacturer's instruction, followed by the calculation of colony numbers and derived total cell numbers 7-10 days later.

Human AML cell lines, such as THP-1 (ATCC), U937 (ATCC) or MV4-11 (ATCC), were cultured in the RPMI-1640 medium containing 10% FBS and subjected to the analysis of cell proliferation changes after *P2X1* knockdown with its specific shRNAs (sh-*P2X1* #1, #2 or scrambled) or after infected with empty vector (EV), WT or mutant *P2X1* (S387A or T389A) plasmids at the indicated time points. For analyzing the proliferation abilities of human cord blood $CD34^+$ cells, the indicated numbers of $CD34^+$ cells were cultured in StemSpan™ SFEM medium (Stemcell Technologies) supplemented with 100 ng/ml SCF, 100 ng/ml TPO and 100 ng/ml FLT3-L. Cell numbers were counted at the indicated time points after culture.

Giemsa-Wright staining and hematoxylin-eosin staining

Wright-Giemsa staining was performed in murine AML cells in blood smear from the peripheral blood from leukemic mice, and the frequencies of blast cells and differentiated leukemic cells were counted according to their typical morphologies. In some cases, livers and spleens of leukemic mice were fixed with 4% paraformaldehyde and embedded in paraffin. Paraffin sections for livers and spleens were subjected to hematoxylin-eosin staining, and the infiltration of AML cells was imaged and evaluated.

Metabolic analyses of oxygen consumption rate, extracellular acidification rate, ATP, TMRM and ROS

FACS-isolated murine AML cells were subjected to the determination in the oxygen consumption rate (OCR) and extracellular acidification rate (ECAR) with XF Cell Mito Stress Test Kit (Agilent, #103015-100) and XF Glycolysis Stress Test Kit (Agilent, #103020-100) using a Seahorse XF96 analyzer according to the manufacturer's instructions. For the detection of mitochondrial stress test, 1.5 μM oligomycin, 2 μM FCCP and 0.5 μM rotenone/antimycin A were injected into port A, B and C, respectively. During the sensor calibration, murine AML cells were incubated in 175 μL of assay medium (XF Base Medium added 2 mM glutamine, 1 mM pyruvate, 10 mM glucose, pH 7.4 at 37 °C) in a 37°C CO_2-free incubator. For the detection of ECAR, the port A on the sensor cartridge was injected with 10 mM glucose. Then, 2.5 μM oligomycin was loaded into port B and 100 mM 2-DG into port C. During sensor calibration, the cells were incubated in 175 μL of assay medium (XF Base Medium supplemented with 1 mM glutamine alone, pH 7.4 at 37 °C) in a 37 °C CO_2-free incubator. ATP levels in LICs were analyzed using ATP Bioluminescence Assay Kit HS II (Roche, #1699709) according to the manufacturer's protocols and data were normalized to the cell counts. Mitochondrial function potential and the levels of reactive oxygen species (ROS) in

LICs were measured with tetramethylrhodamine, methyl ester (TMRM, Sigma-Aldrich, #T5428) and Fluorometric Intracellular Ros deep red Kit (Sigma-Aldrich, #MAK142) following the manufacturer's protocols.

Statistical analysis

Statistical analysis was performed using GraphPad software (Prism 8.0). Data are represented as the mean ± SD unless indicated elsewhere. All the experiments are conducted independently for at least 3 times. Data were analyzed with a Student's t test (two-tailed), one-way ANOVA with Tukey's multiple comparison test, or two-way ANOVA with Sidak's multiple comparison test accordingly. The overall survival of in different groups was compared using the Kaplan-Meier method with a log-rank test. Sample sizes were chosen according to previously performed power analysis. For the animal experiment, a sample size of 5 mice per group and experimental replicate was calculated and used; see figure legends for specific sample sizes, noted as "n". No blinding or randomization was done. Gene expression, cell apoptosis, cell cycle, CFU assay and proliferation experiments were performed in triplicates (3 technical replicates) at least 3 times (3 independent experiments). Chromatin immunoprecipitation and immunoblot experiments were repeated at least 3 times. No data were excluded from analysis. Statistical significance was set at $P<0.05$ (*, $P < 0.05$; **, $P<0.01$; ***, $P<0.001$).

Table S1. Limiting dilution assay for frequencies of functional LICs

**Quantification of CRUs**	**Survival ratio**	
Transplanted cells	WT	KO
100	1/5	3/5
300	0/5	3/5
1000	0/5	1/5
Frequency of leukemia-initiating cells	1:59	1:491
95% confidence interval	(22-157)	(186-877)

Table S2. List of primers and shRNA target sequences

Genotyping primers	Sequences
P2x1-JD313-F	AGCCTTCTTCTTTGAGTATGA
P2x1-JD313-R	ATGCACCCAGAGTAGCG
**q-PCR primers**	**Sequences**
mouse *Alox5*-F	ACTACATCTACCTCAGCCTCATT
mouse *Alox5*-R	GGTGACATCGTAGGAGTCCAC
mouse *Bmi1*-F	ATCCCCACTTAATGTGTGTCCT
mouse *Bmi1*-R	CTTGCTGGTCTCCAAGTAACG
mouse *Cbx5*-F	GACAGGCGCATGGTTAAGG
mouse *Cbx5*-R	CCTGGGCTTATTGTTTTCACCC
mouse *Cebpe*-F	CTGGGGAAGAACAGCTACTTTC
mouse *Cebpe*-R	GTGAGGGATAGGCGAATGGC
mouse *Cebpg*-F	TCGGATCACATTGCTCTGATTTC
mouse *Cebpg*-R	TGTGCCTGAGTATGAATGACACT
mouse *Creb*-F	AGCAGCTCATGCAACATCATC
mouse *Creb*-R	AGTCCTTACAGGAAGACTGAACT
mouse *Gata2*-F	CGACGAGGTGGATGTCTTCT
mouse *Gata2*-R	GCTGTGCAACAAGTGTGGTC
mouse *Gfi1b*-F	ATGCCACGGTCCTTTCTAGTG
mouse *Gfi1b* -R	GGAAGGCTCTGGTTCAGCAA
mouse *Hmgb3*-F	CCCGTCAATTTTGCTGAGTT
mouse *Hmgb3-R*	CCAGGGTTTGTGGATTTGAT
mouse *Hoxb4*-F	CGTGAGCACGGTAAACCCC
mouse *Hoxb4*-R	GTGTTGGGCAACTTGTGGTC

mouse *Klf4*-F	GACTAACCGTTGGCGTGAGG
mouse *Klf4*-R	GTCTAGGTCCAGGAGGTCGT
mouse *Myb*-F	AGAGGGCCATGGGACTAGAT
mouse *Myb*-R	GGGAACGCTTGAGAGTTGAG
mouse *Pu.1*-F	ATGTTACAGGCGTGCAAAATGG
mouse *Pu.1*-R	TGATCGCTATGGCTTTCTCCA
mouse *Runx1*-F	GATGGCACTCTGGTCACCG
mouse *Runx1*-R	GCCGCTCGGAAAAGGACAA
mouse *Stat5b*-F	TGTGGATACAGGCTCAGCAG
mouse *Stat5b*-R	TCAGCAAAAACCCATCTTCC
mouse *Bcat1*-F	GGGCTCAGGATCACAAAGAG
mouse *Bcat1*-R	CAGATCGACCAAGAATGGGT
mouse *Bcat2*-F	TTCATTCGTCAGAGCCTGGATA
mouse *Bcat2*-R	ACTACTCCAGGCAAGATGACGC
mouse *Bckdha*-F	CAGTCCCGCAGGAAGGTGA
mouse *Bckdha*-R	TAGTGCTCCCCGTAGGTCTGC
mouse *Bckdhb*-F	GCAGTGGAACAGGTCCCAGTAG
mouse *Bckdhb*-R	TATCCACATCCCAAGGCACAAT
mouse *Bckdk*-F	GCTTCCGTAGCCTTCCTTT
mouse *Bckdk*-R	GGTGAGTAGCCAGCATTCG
mouse *Ccnt1*-F	AACAAGCGGTGGTATTTTACTCG
mouse *Ccnt1*-R	CCTGCTGGCGGTAAGAGAG
mouse *Dbt*-F	GCTCAGGAAAAGATGGCAGAA
mouse *Dbt*-R	TTTGGGCTGTGGTGGAGGT
mouse *Hoxa9*-F	AAAACACCAGACGCTGGAAC

mouse *Hoxa9*-R	TCTTTTGCTCGGTCCTTGTT
mouse *Meis1*-F	GTTGTCCAAGCCATCACCTT
mouse *Meis1*-R	ATCCACTCGTTCAGGAGGAA
mouse *P2x1*-F	ACTGGGAGTGTGACCTGGAC
mouse *P2x1*-R	TCCCAAACACCTTGAAGAGG
mouse *Pbx1*-F	GAAGAGACGGAATTTCAACAAGC
mouse *Pbx1*-R	CTGTGACAGCCGTTTTGGC
mouse *Pbx3*-F	CGAGGCGCAAGCAAAGAAAC
mouse *Pbx3*-R	TGCCAAAAGCATATTGTCCAGT
mouse *Ppm1k*-F	TCTCATTGGCAAACGGAAAG
mouse *Ppm1k*-R	CAGACAGGTGGGCATAACTCG
mouse *Rora*-F	GTGGAGACAAATCGTCAGGAAT
mouse *Rora*-R	GACATCCGACCAAACTTGACA
mouse β-*actin*-F	GGCTGTATTCCCCTCCATCG
mouse β-*actin*-R	CCAGTTGGTAACAATGCCATGT
human *P2X1*-F	GCTACGTGGTGCAAGAGTCA
human *P2X1*-R	GTAGTTGGTCCCGTTCTCCA
human *BCAT2*-F	CGCTCCTGTTCGTCATTCTCT
human *BCAT2*-R	CCCACCTAACTTGTAGTTGCC
human *BCAT1*-F	GAGCCTGGAAAGGTGGAACTG
human *BCAT1*-R	GCTGACACCCATTATCTACTGCT
human *MEIS1*-F	TACCCGCACACAGCTCATAC
human *MEIS 1*-R	CATTGAATGACTCTGACGAGCA
human *HOXA9*-F	GTCCAAGGCGACGGTGTTT
human *HOXA9*-R	CCGACAGCGGTTCAGGTTTA

human *PBX3*-F	GACGGAAAAGGCGTAACTTCA
human *PBX3*-R	GGTTGCTGAGGTGTGAGTAAAAA
human *PBX1*-F	ATGAATCTCCTGCGAGAGCAA
human *PBX1*-R	CATCCAGAAATCGGGAACGC
human β-*ACTIN*-F	AGAGCTACGAGCTGCCTGAC
human β-*ACTIN*-R	AGCACTGTGTTGGCGTACAG

**shRNAs**	**Target sequences**
Scramble	CCTAAGGTTAAGTCGCCCTCG
mouse sh*P2x1* #1	CCTTTGTAGTTATGACCAATT
mouse sh*P2x1* #2	GCACTACTACAAGCAGAAGAA
human sh*P2X1* #1	TGAAGACGTGTGAGATCTTTG
human sh*P2X1* #2	GTAACCATAGGTGACTAAATT

**Cloning Primers**	**Sequences**
mouse *Bcat1*-XhoI-F	CCGCTCGAGACTGCATCGGCTGCGCTCG
mouse *Bcat1*-EcoRI-R	CCGGAATTCTCAGGGTAGCTCGATTGT
mouse *P2x1*-XhoI-F	CCGCTCGAGAGCTCGGCGGCTGCAGGAT GA
mouse *P2x1*-EcoRI-R	CCGGAATTCTCAGGAGGTCCTCATGTTCT
mouse *P2x1(S387A)*-EcoRI-R	CGGAATTCTCAGGAGGTCCTCATGTTCTC CTGCAGGCCCAGAGTGGAGgcGGTGGC
mouse *P2x1(S388A)*-EcoRI-R	CGGAATTCTCAGGAGGTCCTCATGTTCTC CTGCAGGCCCAGAGTGGcGCT
mouse *P2x1(T389A)*-EcoRI-R	CGGAATTCTCAGGAGGTCCTCATGTTCTC CTGCAGGCCCAGcGcGGAGCT
mouse *Pbx3*-BamHI-F	CGCGGATCCATGGACGATCAATCCAGG

mouse *Pbx3*-NotI-R	ATAAGAATGCGGCCGCAGTTAGAGGTATC CGA
human *P2X1*-EcoRI-F	CCGGAATTCATGGCACGGCGGTTCCAG
human *P2X1*-Xba1-R	GCTCTAGAGGATGTCCTCATGTTCTC
human *P2X1(S387A)*-Xba1-R	GCTCTAGAGGATGTCCTCATGTTCTCCTCA TGTTCTCCTGCAGGCCCAGGGTGGAGgcG GTAGCT
human *P2X1(T389A)*-Xba1-R	GCTCTAGAGGATGTCCTCATGTTCTCCTCA TGTTCTCCTGCAGGCCCAGGGcGGAGCT

**Luciferase primers**	**Sequences**
mouse *Bcat1*-promoter-F	CCTGAGCTCGCTAGCCTCGAGACACACAC ACACACACACAC
mouse *Bcat1*-promoter-R	CAGTACCGGATTGCCAAGCTTCTGGCGGG CCAGGGTTGCAG
Bcat1-F (for ChIP)	GTGCAATGTATGGGTTCC
Bcat1-R (for ChIP)	TCTGGCCTTGACCTCTCC
