# Supplementary material for: P2X1 enhances leukemogenesis through PBX3-BCAT1 pathways
Source: Leukemia. 2022 Nov 23;37(2):265–75. doi: 10.1038/s41375-022-01759-y (PMC9898031; doi:10.1038/s41375-022-01759-y)
Supplement: Supplementary file 2 — SUPPLEMENTARY MATERIAL [file 41375_2022_1759_MOESM2_ESM.docx]

**Table S2. List of primers and shRNA target sequences**

| **Genotyping primers** | **Sequences** |
| --- | --- |
| *P2x1*-JD313-F | AGCCTTCTTCTTTGAGTATGA |
| *P2x1*-JD313-R | ATGCACCCAGAGTAGCG |
| **q-PCR primers** | **Sequences** |
| mouse *Alox5*-F | ACTACATCTACCTCAGCCTCATT |
| mouse *Alox5*-R | GGTGACATCGTAGGAGTCCAC |
| mouse *Bmi1*-F | ATCCCCACTTAATGTGTGTCCT |
| mouse *Bmi1*-R | CTTGCTGGTCTCCAAGTAACG |
| mouse *Cbx5*-F | GACAGGCGCATGGTTAAGG |
| mouse *Cbx5*-R | CCTGGGCTTATTGTTTTCACCC |
| mouse *Cebpe*-F | CTGGGGAAGAACAGCTACTTTC |
| mouse *Cebpe*-R | GTGAGGGATAGGCGAATGGC |
| mouse *Cebpg*-F | TCGGATCACATTGCTCTGATTTC |
| mouse *Cebpg*-R | TGTGCCTGAGTATGAATGACACT |
| mouse *Creb*-F | AGCAGCTCATGCAACATCATC |
| mouse *Creb*-R | AGTCCTTACAGGAAGACTGAACT |
| mouse *Gata2*-F | CGACGAGGTGGATGTCTTCT |
| mouse *Gata2*-R | GCTGTGCAACAAGTGTGGTC |
| mouse *Gfi1b*-F | ATGCCACGGTCCTTTCTAGTG |
| mouse *Gfi1b* -R | GGAAGGCTCTGGTTCAGCAA |
| mouse *Hmgb3*-F | CCCGTCAATTTTGCTGAGTT |
| mouse *Hmgb3*-R | CCAGGGTTTGTGGATTTGAT |
| mouse *Hoxb4*-F | CGTGAGCACGGTAAACCCC |
| mouse *Hoxb4*-R | GTGTTGGGCAACTTGTGGTC |
| mouse *Klf4*-F | GACTAACCGTTGGCGTGAGG |
| mouse *Klf4*-R | GTCTAGGTCCAGGAGGTCGT |
| mouse *Myb*-F | AGAGGGCCATGGGACTAGAT |
| mouse *Myb*-R | GGGAACGCTTGAGAGTTGAG |
| mouse *Pu.1*-F | ATGTTACAGGCGTGCAAAATGG |
| mouse *Pu.1*-R | TGATCGCTATGGCTTTCTCCA |
| mouse *Runx1*-F | GATGGCACTCTGGTCACCG |
| mouse *Runx1*-R | GCCGCTCGGAAAAGGACAA |
| mouse *Stat5b*-F | TGTGGATACAGGCTCAGCAG |
| mouse *Stat5b*-R | TCAGCAAAAACCCATCTTCC |
| mouse *Bcat1*-F | GGGCTCAGGATCACAAAGAG |
| mouse *Bcat1*-R | CAGATCGACCAAGAATGGGT |
| mouse *Bcat2*-F | TTCATTCGTCAGAGCCTGGATA |
| mouse *Bcat2*-R | ACTACTCCAGGCAAGATGACGC |
| mouse *Bckdha*-F | CAGTCCCGCAGGAAGGTGA |
| mouse *Bckdha*-R | TAGTGCTCCCCGTAGGTCTGC |
| mouse *Bckdhb*-F | GCAGTGGAACAGGTCCCAGTAG |
| mouse *Bckdhb*-R | TATCCACATCCCAAGGCACAAT |
| mouse *Bckdk*-F | GCTTCCGTAGCCTTCCTTT |
| mouse *Bckdk*-R | GGTGAGTAGCCAGCATTCG |
| mouse *Ccnt1*-F | AACAAGCGGTGGTATTTTACTCG |
| mouse *Ccnt1*-R | CCTGCTGGCGGTAAGAGAG |
| mouse *Dbt*-F | GCTCAGGAAAAGATGGCAGAA |
| mouse *Dbt*-R | TTTGGGCTGTGGTGGAGGT |
| mouse *Hoxa9*-F | AAAACACCAGACGCTGGAAC |
| mouse *Hoxa9*-R | TCTTTTGCTCGGTCCTTGTT |
| mouse *Meis1*-F | GTTGTCCAAGCCATCACCTT |
| mouse *Meis1*-R | ATCCACTCGTTCAGGAGGAA |
| mouse *P2x1*-F | ACTGGGAGTGTGACCTGGAC |
| mouse *P2x1*-R | TCCCAAACACCTTGAAGAGG |
| mouse *Pbx1*-F | GAAGAGACGGAATTTCAACAAGC |
| mouse *Pbx1*-R | CTGTGACAGCCGTTTTGGC |
| mouse *Pbx3*-F | CGAGGCGCAAGCAAAGAAAC |
| mouse *Pbx3*-R | TGCCAAAAGCATATTGTCCAGT |
| mouse *Ppm1k*-F | TCTCATTGGCAAACGGAAAG |
| mouse *Ppm1k*-R | CAGACAGGTGGGCATAACTCG |
| mouse *Rora*-F | GTGGAGACAAATCGTCAGGAAT |
| mouse *Rora*-R | GACATCCGACCAAACTTGACA |
| mouse β-*actin*-F | GGCTGTATTCCCCTCCATCG |
| mouse β-*actin*-R | CCAGTTGGTAACAATGCCATGT |
| human *P2X1*-F | GCTACGTGGTGCAAGAGTCA |
| human *P2X1*-R | GTAGTTGGTCCCGTTCTCCA |
| human *BCAT2*-F | CGCTCCTGTTCGTCATTCTCT |
| human *BCAT2*-R | CCCACCTAACTTGTAGTTGCC |
| human *BCAT1*-F | GAGCCTGGAAAGGTGGAACTG |
| human *BCAT1*-R | GCTGACACCCATTATCTACTGCT |
| human *MEIS1*-F | TACCCGCACACAGCTCATAC |
| human *MEIS 1*-R | CATTGAATGACTCTGACGAGCA |
| human *HOXA9*-F | GTCCAAGGCGACGGTGTTT |
| human *HOXA9*-R | CCGACAGCGGTTCAGGTTTA |
| human *PBX3*-F | GACGGAAAAGGCGTAACTTCA |
| human *PBX3*-R | GGTTGCTGAGGTGTGAGTAAAAA |
| human *PBX1*-F | ATGAATCTCCTGCGAGAGCAA |
| human *PBX1*-R | CATCCAGAAATCGGGAACGC |
| human β-*ACTIN*-F | AGAGCTACGAGCTGCCTGAC |
| human β-*ACTIN*-R | AGCACTGTGTTGGCGTACAG |
| **shRNAs** | **Target sequences** |
| Scramble | CCTAAGGTTAAGTCGCCCTCG |
| mouse sh*P2x1* #1 | CCTTTGTAGTTATGACCAATT |
| mouse sh*P2x1* #2 | GCACTACTACAAGCAGAAGAA |
| human sh*P2X1* #1 | TGAAGACGTGTGAGATCTTTG |
| human sh*P2X1* #2 | GTAACCATAGGTGACTAAATT |
| **Cloning Primers** | **Sequences** |
| mouse *Bcat1*-XhoI-F | CCGCTCGAGACTGCATCGGCTGCGCTCG |
| mouse *Bcat1*-EcoRI-R | CCGGAATTCTCAGGGTAGCTCGATTGT |
| mouse *P2x1*-XhoI-F | CCGCTCGAGAGCTCGGCGGCTGCAGGATGA |
| mouse *P2x1*-EcoRI-R | CCGGAATTCTCAGGAGGTCCTCATGTTCT |
| mouse *P2x1(S387A)*-EcoRI-R | CGGAATTCTCAGGAGGTCCTCATGTTCTCCTGCAGGCCCAGAGTGGAGgcGGTGGC |
| mouse *P2x1(S388A)*-EcoRI-R | CGGAATTCTCAGGAGGTCCTCATGTTCTCCTGCAGGCCCAGAGTGGcGCT |
| mouse *P2x1(T389A)*-EcoRI-R | CGGAATTCTCAGGAGGTCCTCATGTTCTCCTGCAGGCCCAGcGcGGAGCT |
| mouse *Pbx3*-BamHI-F | CGCGGATCCATGGACGATCAATCCAGG |
| mouse *Pbx3*-NotI-R | ATAAGAATGCGGCCGCAGTTAGAGGTATCCGA |
| human *P2X1*-EcoRI-F | CCGGAATTCATGGCACGGCGGTTCCAG |
| human *P2X1*-Xba1-R | GCTCTAGAGGATGTCCTCATGTTCTC |
| human *P2X1(S387A)*-Xba1-R | GCTCTAGAGGATGTCCTCATGTTCTCCTCATGTTCTCCTGCAGGCCCAGGGTGGAGgcGGTAGCT |
| human *P2X1(T389A)*-Xba1-R | GCTCTAGAGGATGTCCTCATGTTCTCCTCATGTTCTCCTGCAGGCCCAGGGcGGAGCT |
| **Luciferase primers** | **Sequences** |
| mouse *Bcat1*-promoter-F | CCTGAGCTCGCTAGCCTCGAGACACACACACACACACACAC |
| mouse *Bcat1*-promoter-R | CAGTACCGGATTGCCAAGCTTCTGGCGGGCCAGGGTTGCAG |
| *Bcat1*-F (for ChIP) | GTGCAATGTATGGGTTCC |
| *Bcat1*-R (for ChIP) | TCTGGCCTTGACCTCTCC |
